# Development of a probabilistic early health warning system based on meteorological parameters

**DOI:** 10.1038/s41598-020-71668-6

**Published:** 2020-09-08

**Authors:** A. K. Sahai, Raju Mandal, Susmitha Joseph, Shubhayu Saha, Pradip Awate, Somenath Dutta, Avijit Dey, Rajib Chattopadhyay, R. Phani, D. R. Pattanaik, Sunil Despande

**Affiliations:** 1 Indian Institute of Tropical Meteorology, Dr. Homi Bhabha Road, Pashan, Pune, 411 008 India; 2 Department of Atmospheric and Space Sciences, Savitribai Phule Pune University, Pune, India; 3 Rollins School of Public Health, Emory University, Atlanta, GA USA; 4 Integrated Disease Surveillance Program, Pune, Maharashtra India; 5 India Meteorological Department (IMD), Pune, India; 6 India Meteorological Department (IMD), New Delhi, India

**Keywords:** Climate sciences, Atmospheric science, Climate change, Environmental sciences, Environmental impact

## Abstract

Among the other diseases, malaria and diarrhoea have a large disease burden in India, especially among children. Changes in rainfall and temperature patterns likely play a major role in the increased incidence of these diseases across geographical locations. This study proposes a method for probabilistic forecasting of the disease incidences in extended range time scale (2–3 weeks in advance) over India based on an unsupervised pattern recognition technique that uses meteorological parameters as inputs and which can be applied to any geographical location over India. To verify the robustness of this newly developed early warning system, detailed analysis has been made in the incidence of malaria and diarrhoea over two districts of the State of Maharashtra. It is found that the increased probabilities of high (less) rainfall, high (low) minimum temperature and low (moderate) maximum temperature are more (less) conducive for both diseases over these locations, but have different thresholds. With the categorical probabilistic forecasts of disease incidences, this early health warning system is found to be a useful tool with reasonable skill to provide the climate-health outlook about possible disease incidence at least 2 weeks in advance for any location or grid over India.

## Introduction

The adverse health burden associated with malaria and diarrhoea is significant in India, especially among children^1–6^. There are various complex ways in which the weather parameters (e.g. temperature, precipitation, humidity etc.) can directly or indirectly affect the incidence of these diseases. Changes in local weather patterns alter the ecological conditions which can affect vector ecology and then indirectly impacting human health^7^.

Temporal and spatial variability in the weather parameters, for example, a short-term increase in temperature and rainfall as an effect of El-Niño can lead to malaria epidemics^8^. On the other hand, very high temperatures that are beyond the physiological tolerance limit of the parasite can kill the parasite, thereby decreasing malaria transmission. However, at lower temperatures (climatological), a small increase in temperature can significantly increase the risk of malaria transmission due to the increased numbers of mosquitoes^9^. In some areas, heavy rainfall events can wash out the breeding sites, resulting in a reduction in the incidence of malaria. A study^10^ has proposed a set of transmission windows for Indian region, in terms of different temperature ranges for a particular range of relative humidity and found that, the malaria-prone regions may shift from central Indian regions to the south-western and northern States by the 2050s and it is likely to persist over north-eastern parts of the country. Using a dynamical model for malaria transmission, a recent study has identified that a particular range of rainfall (~ 200–360 mm/month) and temperature (monthly mean of ~ 28–29 $\mathrm{^\circ{\rm C} })$ are favourable for malaria outbreaks over the State of Odisha, India mainly during monsoon months^11^. Another study^12^ has evaluated the impacts of climatic parameters (maximum and minimum temperatures and rainfall) on malaria epidemics and the effectiveness of malaria control during the epidemics in India. It has been found that malaria epidemics mostly occurred in summer and autumn seasons. There is an evidence of using other parameters like topography and vegetation along with the climatic variables (temperature, relative humidity, rainfall and soil) into the framework of malaria risk modelling to identify the malaria risk zones in India^13^.

The Intergovernmental Panel on Climate Change noted in its 2007 report that climate change may increase the risk of diarrhoeal diseases^14^, which is of major concern in the developing countries^15^. Previous studies^16–18^ documented that various weather parameters play a major role in the incidence of diarrhoeal disease. Studies illustrated that after a flood-event^19,20^ or heavy rainfall^17,21^, the rate of incidence of diarrhoeal disease may increase, especially in the areas with poor sanitation facilities. Again, drought conditions can reduce the availability of fresh water and can lead to diarrhoeal and other diseases due to poor hygiene^22^. Other studies^23,24^ observed that the occurrence of diarrhoeal diseases was maximum during the summer months followed by rainy or winter months over two slum areas in Pondicherry and Delhi. These studies put forward the requirement of identifying the specific combination of local weather parameters at different locations in generating ideal conditions for the spread of the disease.

Therefore, forecasting potential cases of such diseases is critical for early health planning and disease prevention. Traditional time series forecasting methods (autoregressive integrated moving average (ARIMA) model and seasonal ARIMA (SARIMA) model) have been used for infectious diseases, but they capture only the linear trend in the data. These models also require a priori knowledge of the data-generating process to correctly specify the seasonality in the data which may not be known. Besides, the Bayesian approach (statistical probabilistic approach) forces the analyst to look at historical data sets. The process of choosing prior distributions can be very time-consuming and frustrating. As an alternative, models based on neural networks are designed to identify complex non-linear relationships in the data. Since the literature strongly suggest non-linear associations between the meteorological parameters (temperature, rainfall) and health outcomes (malaria, diarrhoea), the application of neural network is particularly appealing in such studies. The ability of this modelling framework to parse huge datasets into several smaller batches and train the network through multiple stages helps in the identification of spatial and temporal patterns that the other methods do not offer. A study^25^ has compared the performances of SARIMA model and three different models which are based on Artificial Neural Networks (namely Back-propagation neural networks, Radial Basis Function Neural Networks and Elman recurrent neural networks) for the forecasting of Typhoid. It showed that any of the three models based on neural networks performed better than SARIMA model in both the modelling and forecasting processes for Typhoid Fever Incidence in China based on 6 years data. Also, there are many reviews of epidemiology models which are ordinary differential equation models and mainly used where individuals infect each other directly (rather than through a disease vector such as a mosquito). A complete list of them is available in the study by Mutalik AV, 2017 and references 9–38 therein^26^. Very commonly used among all of them are susceptible–infectious–recovered (SIR)^27^ and susceptible-exposed-infectious-recovered (SEIR) models which are basically deterministic and compartmental models. A very common practice when working with these models is model calibration to adjust the model parameters until the model output closely matches empirical data. For this purpose one need to have prior information about the observed disease data. They use the observed disease data as different compartments such as the number of susceptible, infected and recovered cases per day or per week and so on. Since our main goal in this study is to develop a probabilistic early health warning system (hereafter EHWS) based on meteorological parameters, we have chosen a neural network based forecasting method that doesn’t require any prior information about the input variables.

Since the ranges of different climatic parameters differ across geographical regions, location-specific assessments of the association between these meteorological parameters and health outcomes need to be conducted to inform the design of EHWS. This study uses historical data to assess the association between malaria (hereafter MAL) and acute diarrheal diseases (hereafter ADD) and the meteorological parameters for two cities (Pune and Nagpur; hereafter PNE and NGP respectively) in India using a technique based on unsupervised learning neural networks i.e. Self Organizing Map (SOM)^28,29^. An outline is provided on how a combination of the weather-health information derived in conjunction with the real-time extended range climatic forecasts can inform public health action through the development of EHWS 2–3 weeks in advance. In this study two SOM clustering have been performed; (i) 3 × 3 clustering, used for easy visualization of the capabilities of SOM method and (ii) 6 × 6 clustering, used for real forecasting. This communication also emphasises that the use of longer period and pan India health data is prerequisite for the development of skilful and robust EHWS and therefore, data sharing between health and climate researchers is essential. However, the main constrain is to get the health data from Public Health Department, Govt. of India. We hope that the results of this paper may encourage more strong collaboration between health and climate researchers for the improvement of the EHWS.

## Results

### Observed cases of MAL and ADD

**Figure 1:**
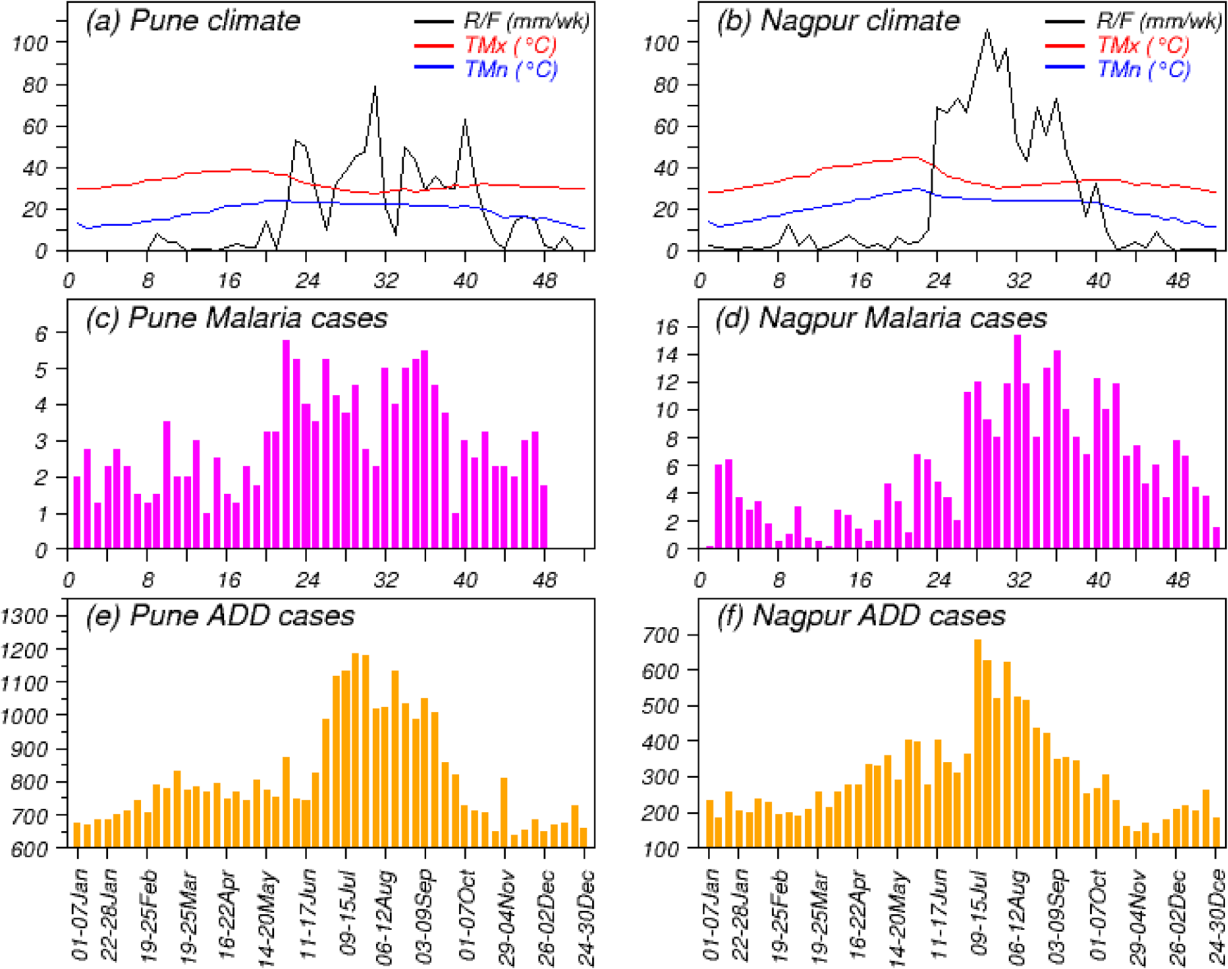
Observed climatological weekly average: (**a**) and (**b**) R/F, TMx and TMn; (**c**) and (**d**) number of MAL cases; and (**e**) and (**f**) number of ADD cases over Pune and Nagpur respectively. The scales on Y-axes are same for R/F, TMx and TMn in (**a**) and (**b**).

The actual recorded weekly mean of MAL and ADD cases along with the climatological rainfall (hereafter R/F), maximum temperature (hereafter TMx) and minimum temperature (hereafter TMn) over PNE and NGP regions are shown in Fig. 1 (X axes represent the weeks of a calendar year and Y axes for R/F or TMx or TMn or average cases of MAL or ADD). The Fig. 1a and b represent climatological values of R/F, TMx and TMn over PNE and NGP respectively. It is seen that rainy season over PNE lasts from June to mid-October, whereas over NGP it lasts for June–September (JJAS). TMx and Tmin are higher over both the regions during pre-monsoon months of March–May and drop down during monsoon months (JJAS). Figure 1c and d show the MAL cases and Fig. 1e and f show the ADD cases over PNE and NGP respectively. It is found that NGP experiences more MAL cases as compared to PNE, whereas PNE experiences more ADD cases compared to NGP (as per record). PNE experiences more or less constant number of ADD cases during all seasons except an increase during Mid-June to September, whereas over NGP the magnitude of ADD cases is more during July to August months as compared to other seasons. MAL cases are observed more before and during monsoon season over PNE. In contrast, the MAL cases are more after one month of monsoon onset to post monsoon month over NGP. This type of variability in magnitudes and duration may be attributed to the different geographical positions and different climatic conditions (as seen in Fig. 1a and b) of these two places. So, the variation in R/F distribution, variation in TMx and TMn over these two regions may play a major role behind the seasonality of the MAL and ADD incidences.

### Mean map obtained from 3 × 3 SOM clustering

**Figure 2:**
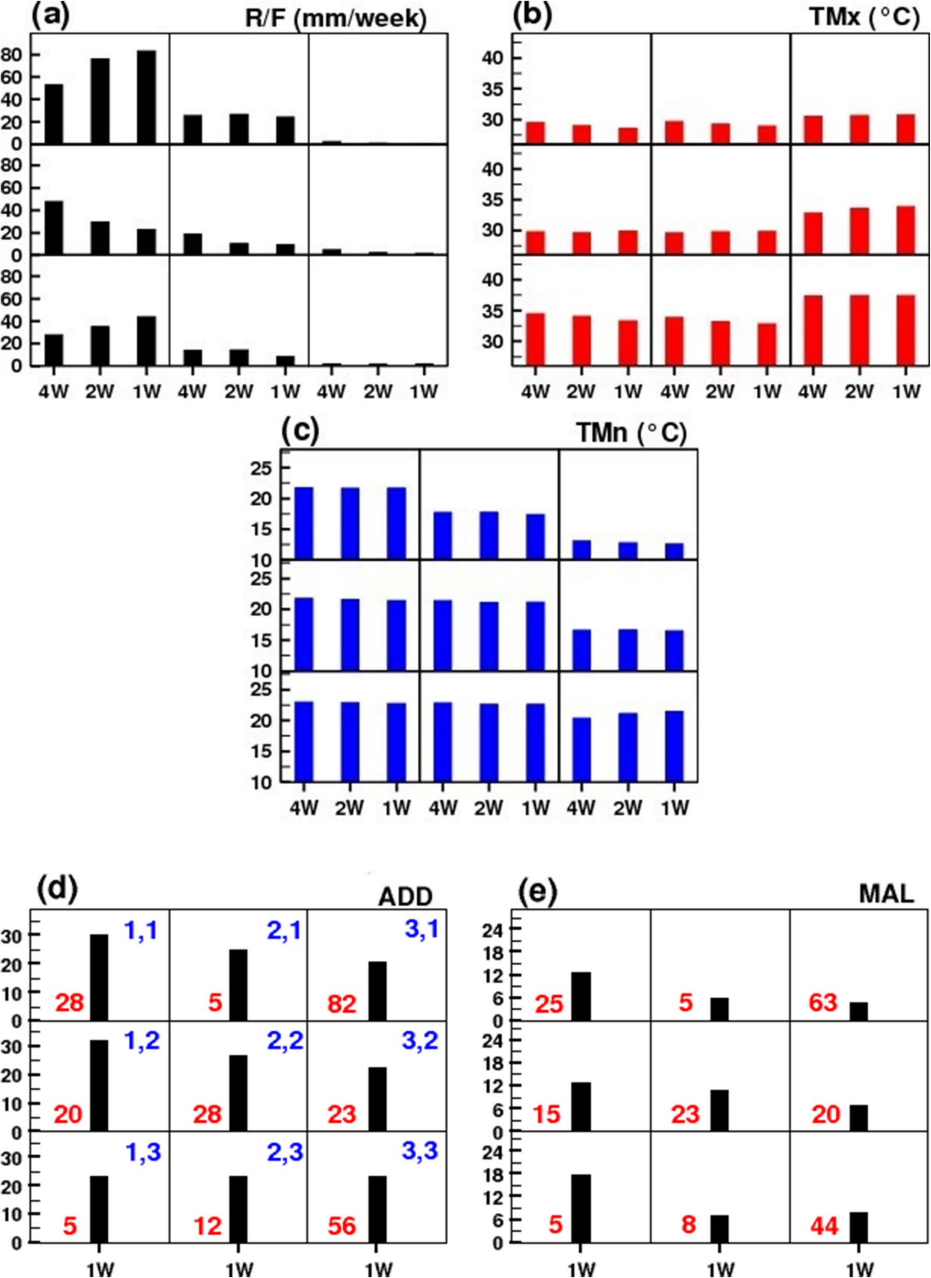
Mean map of all the cases clustered in each of 9 nodes obtained from 3 × 3 SOM clustering for Pune: (**a**) R/F, (**b**) TMx, (**c**) TMn and (**d**) ADD cases (per 1,00,000 populations) and (**e**) MAL cases (per 10 million populations). Here, the positions of the nodes are mentioned on right-top of the each sub-panel of (**d**) and the numbers of cases are mentioned in red colour for ADD and MAL (in **d** and **e**).

Figure 2 shows the mean map obtained from all cases clustered in individual node for R/F, TMx, TMn and MAL for PNE. In 9 sub-panels (corresponds to each individual node) of Fig. 2a–c, the mean values for 1 W (current week), 2 W (average of current week and previous week) and 4 W (average of current week and last three weeks) of R/F, TMx and TMn are plotted. Figure 2d and e represent average cases of ADD (per 1,00,000 populations) and MAL (per 10 Million populations) in each node only for 1 W (i.e. current week) (please refer Data and Methods section) respectively. The positions of the nodes are mentioned on the right-top of each sub-panel (Fig. 2d) by the notation x,y and the numbers of clustered cases are also mentioned in red colour for ADD and MAL (Fig. 2d and e). From Fig. 2d,e, it is observed that, the higher valued ADD cases are clustered in nodes (1,1) and (1,2) and higher valued MAL cases are in node (1,3), while the minimum valued cases are clustered in node (3,1) for both ADD and MAL. It is seen that the large number of ADD and MAL cases are associated with moderate to heavy R/F activities, higher TMn and moderate TMx values in all 1w, 2w and 4w weeks. It is noted that the highest cases of ADD (node (1,2)) is associated with (i) the highest amount of R/F during week 4 W (48 mm/week) and with decreasing amount from week 4 W to 1 W (23 mm/week), (ii) relatively high values of TMn ($\sim 21.5^\circ{\rm C}$) during all 4 weeks and (iii) moderate TMx values ($\sim 30^\circ{\rm C}$) during all 4 weeks. On the other hand, highest cases of MAL (node (1,3) is associated with (i) the highest amount of R/F during current week 1 W (44 mm/week) and with decreasing amount from week 1 W to 4 W (27 mm/week), (ii) relatively high values of TMn ($\sim 23^\circ{\rm C}$) during all 4 weeks and (iii) moderate to high TMx values ($\sim 34^\circ{\rm C}$) during all 4 weeks. The least number of ADD and MAL cases (in node (3,1)) can be linked with very dry condition, moderate TMx ($\sim 31^\circ{\rm C}$) and low TMn ($\sim 13^\circ{\rm C}$) values. So, it can be concluded that the thresholds of climatic variables for the outbreaks are different for different diseases over PNE.

The similar mean map for NGP region is obtained (please refer Supplementary Figure S2) and it found that over this region the highest number of ADD and MAL cases (node (1,1)) are associated with the heavy R/F activities in all 4 weeks (range: 93–104 mm/week), high TMn ($\sim 24^\circ{\rm C} )$ and moderate TMx values ($range: 31-32^\circ{\rm C} )$ in all 4 weeks. The lowest ADD cases (node (3,1)) are observed to link with no rain, low TMn ($\sim 15^\circ{\rm C} )$ and moderate TMx ($\sim 31^\circ{\rm C} )$. Whereas the lowest cases of MAL (node (3,3)) are linked with no rain, very high TMx ($\sim 42^\circ{\rm C} )$ and considerably high Tmn ($\sim 25-26^\circ{\rm C} )$. Therefore, it is found that over these two regions the outbreaks and lowest cases of ADD and MAL are strongly linked to the different thresholds of weather parameters, i.e., on different weather conditions.

The actual distributions of these 3 weather parameters and 2 diseases around the mean in different nodes are presented through the Box-and-Whisker diagrams^30^ in Supplementary Information by Figure S3 and S4 for PNE and NGP respectively.

### Class probabilities obtained from 3 × 3 SOM clustering

**Figure 3:**
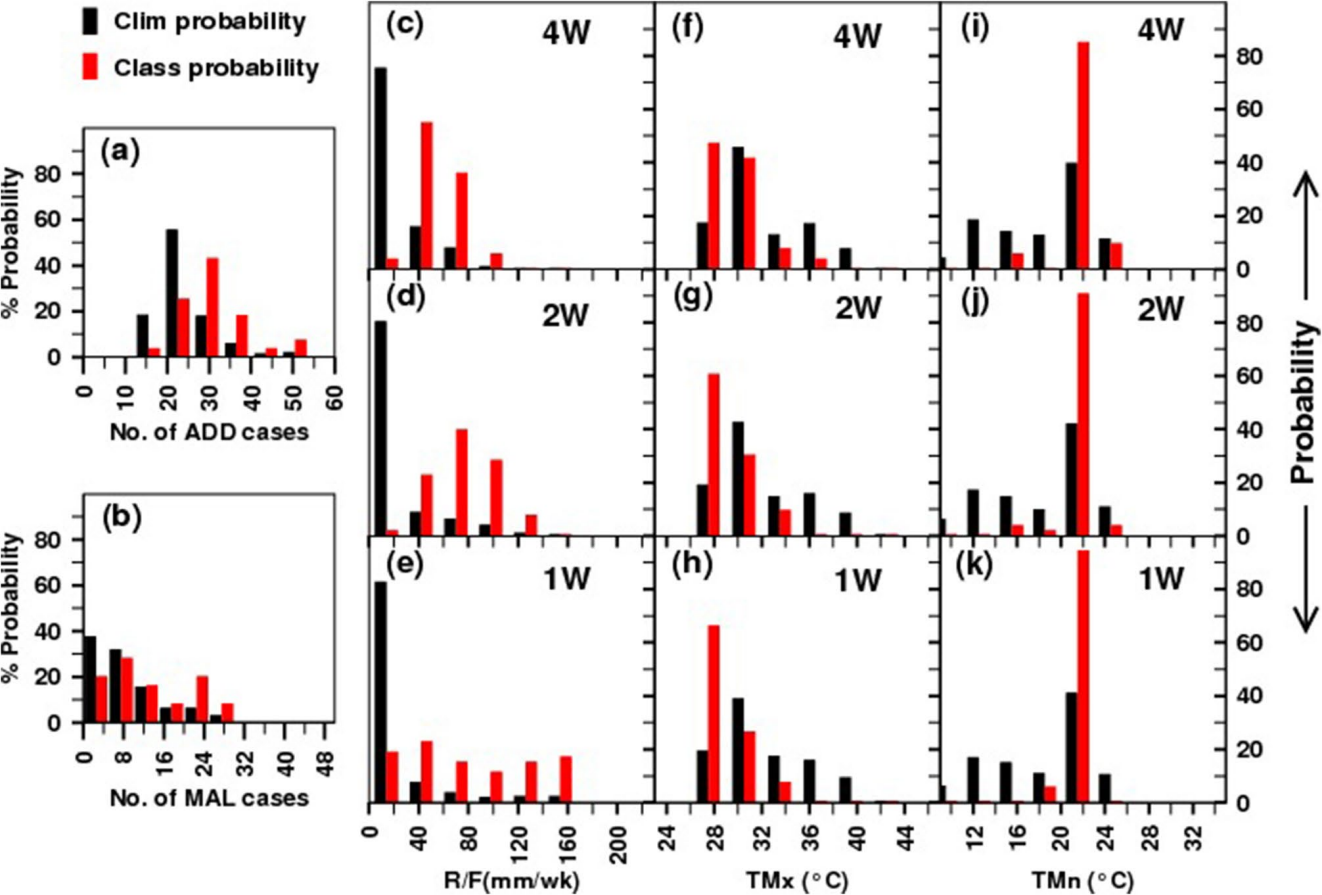
Class probabilities (red bars) with climatological probabilities (black bars) of: (**a**) ADD; (**b**) MAL; (**c**)–(**e**) R/F for 1w, 2w and 4w time steps; (**f**)–(**h**) TMx for 1w, 2w and 4w time steps and (**i**)–(**k**) TMn for 1w, 2w and 4w time steps for node (1,1) and for Pune.

The class probabilities (red bars) along with the climatological probabilities (black bars) of three weather parameters and the disease cases are shown in Fig. 3 for the node (1,1) (as an example) for PNE, where the number of ADD and MAL cases are found larger. Class probability means the probability of getting a certain value in that particular node among the cases clustered into that node, whereas the climatological probability represents the same but considering all the 9 nodes together. The class probabilities and the climatological probabilities of getting different number of ADD and MAL cases are presented in Fig. 3a and b respectively. Similarly the same probabilities for R/F, TMx and TMn are plotted in panel c-e, f–h and i-k respectively with different time steps i.e. 4 W, 2 W and 1 W. It is found that the increased probabilities of wet spell, high TMn and moderate TMx are more conducive for large number of ADD and MAL cases (Fig. 3a–k). Similarly from the class probabilities of node (3,1) (please refer Supplementary Figure S5), it is observed that, the increased probabilities of dry spell, low TMn and moderate TMx are less conducive for ADD and MAL over PNE. Also, from the probabilistic analysis for NGP region (please refer Supplementary Figure S6-7), it is found that the increased probabilities of heavy to very heavy R/F (less R/F), high to very high TMn (low to medium TMn) and low TMx (moderate to high TMx) are more (less) conducive for ADD and MAL over this region.

### Skill of the 6 × 6 SOM based EHWS

Before implementing the real-time prediction of the disease incidences, it is essential to check the skill of this EHWS. To evaluate the skill, the Correlation Coefficient (CC), Root Mean Square Error (RMSE) and Brier Skill Scores (BSS)^31^ for different categorical probabilistic forecasts are calculated with respect to the actual observations for MAL and ADD over the two regions separately. To calculate the CC and RMSE, the deterministic forecasts are used after removing the climatological bias (also the CC and RMSE values without bias correction are placed in Supplementary Table S2). The CC, RMSE and BSS for Below Normal (BN), Near Normal (NN) and Above Normal (AN) (please refer Data and Methods section) along with the climatological skill scores (presented inside the brackets) are listed in Table 1 for 6 × 6 SOM analysis. It can be noticed that for the prediction system, the CC values for MAL and ADD over these two regions are ~ 0.7 or more and also the RMSE values are reasonable i.e. ~ 5 or less cases (except for NGP MAL i.e. ~ 15). Also, we can see that the CC and RMSE values for the model are better than the same calculated from climatological forecasts. For the perfect forecast, the value of BSS is 1 and for no skill it is 0 and if it is negative, then the forecast quality is poorer than the climatology. The positive value indicates good forecast quality, so higher is the value more improvement in the forecast compared to the climatology. From the table it is seen, the BSS values are all positive for all BN, NN and AN categories and showing higher skills as compared to the climatology over both the regions and for both MAL and ADD. Thus, analysis of the skill scores indicates that the EHWS always exhibits better forecasting ability than the climatology, and it is much better for probabilistic forecasts.

**Table 1:** Correlation coefficient (CC) and root mean square error (RMSE) between observed and predicted Malaria and ADD cases and brier skill scores (BSS) between observed and predicted probabilities for different categories such as below normal (BN), near normal (nn) and above normal (AN). The climatological skill scores are also provided inside the brackets.

Type of events	CC model (clim)	RMSE model (clim)	BSS		
			BN (clim)	NN (clim)	AN (clim)
**6 × 6 SOM**					
Pune MAL	0.68 (0.57)	5.6 (6.3)	0.21 (− 0.12)	0.14 (− 1.01)	0.21 (–0.33)
Nagpur MAL	0.74 (0.71)	15.4 (16.2)	0.21 (0.05)	0.15 (− 0.41)	0.29 (0.07)
Pune ADD	0.72 (0.68)	4.7 (5.0)	0.17 (− 0.28)	0.21 (− 0.68)	0.38 (0.22)
Nagpur ADD	0.72 (0.72)	4.8 (4.8)	0.31 (− 0.002)	0.14 (− 0.63)	0.41 (− 0.23)

So, the above skill analysis gives us the confidence to use this EHWS based on 6 × 6 SOM techniques for the real-time prediction of such disease incidences over any other places of India.

### Prediction and verification of disease incidences

**Figure 4:**
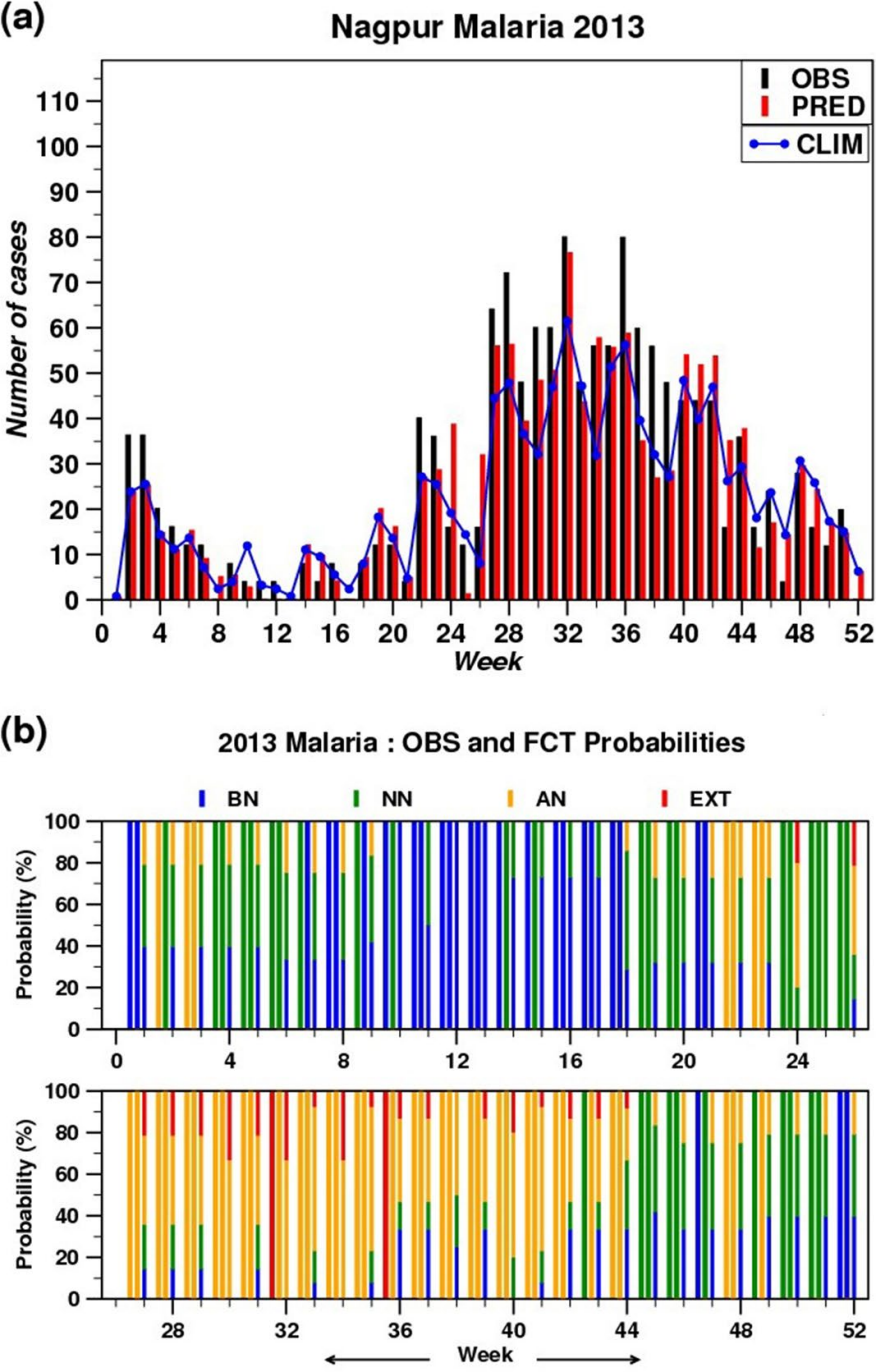
(**a**) Weekly observed values (black bars), predicted values (red bars) and the climatology (blue line); and (**b**) probabilistic forecasts: % probabilities of Below Normal (BN), Near Normal (NN), Above Normal (AN) and Extreme (EXT) occurrences for MAL during 2013 over Nagpur. The left most bar represents the observed probability, middle bar represents the climatological probability and right most bar is for the forecast probability. For observation and climatology any of the above probabilities is 100%.

The weekwise deterministic as well as the categorical probabilistic forecasts of MAL and ADD cases are produced during the analysis period (2009–16) for all the years and for both the regions by using 6 × 6 SOM technique and weather parameters as predictors. Figure 4a shows the weekly actual recorded cases (black bars), the deterministic predicted (bias-corrected) values (red bars) and the climatology (blue line) of MAL during year 2013 for NGP as an example. From Fig. 4a, it can be noted that the variability of the occurrence of MAL over this region over different season is nicely captured by the SOM based EHWS. Though, it failed to predict the actual number of cases that occurred in a few occasions. For example, the predicted number of MAL cases during few weeks are either much less (e.g. week no. 2, 3, 22, 23, 25, 27, 28, 36, 37, 38 and 39) or much higher (e.g. week no. 19, 24, 26, 43, 47 and 49) as compared to the actual observed cases.

For the same year, the probabilistic forecasts i.e. percentage probabilities of BN, NN, AN and extreme occurrences (EXT) (please refer Data and Methods section) of MAL for NGP is shown in Fig. 4b. For each week, the left most bar represents the observed probability, middle bar represents the climatological probability and right most bar is for the forecast probability. For observation and climatology any of the above probabilities is 100%. The forecasted probability contains four categories as mentioned above and the % probability for each category is shown with the length of the four segments in the right most bar with different colours (red, yellow, green and blue for EXT, AN, NN and BN respectively). The significance of this plot can be explained in a much easier way with the help of Fig. 4a. For example, in Fig. 4a at week numbers 19 and 47 the prediction is more than the actual observation and the differences are high. However, it is observed from Fig. 4b that during these weeks the probabilistic forecasts with four different categories is providing much more realistic information i.e. showing (zero for Extreme and ~ 15% for above normal) more probabilities for NN and BN categories, against the observed probabilities of BN categories. Again for week numbers 24, 26, 43 and 49 where deterministic forecasts are much higher compared to actual, the probabilistic forecasts show high probability for NN and AN against NN categories in observations. There are other evidences where actual observation is large but the deterministic prediction is much on the lower side e.g. week numbers 2, 3, 22, 23 and 38. The categorical forecasts during the same weeks show reasonable probabilities of NN and AN against observed probabilities of AN. Again for week numbers 32 and 36 (where observed probabilities are EXT), the forecasts show high probabilities for AN with less probability of EXT categories.

The same analysis is done for other remaining years for MAL and ADD for both the regions and selective years are kept as the supplementary information (Figure S8-15). From the analysis of all deterministic and probabilistic forecasts, it can be noted that, the SOM based method could nicely capture the seasonal variability of occurrence of the diseases over these two different geographical locations, although it failed to predict the exact magnitudes in a few occasions. Also, it is found that the categorical probabilistic forecasts can provide more realistic and scientific information which can be very useful for policy making than the deterministic forecasts.

### Real-time extended range forecast

**Figure 5:**
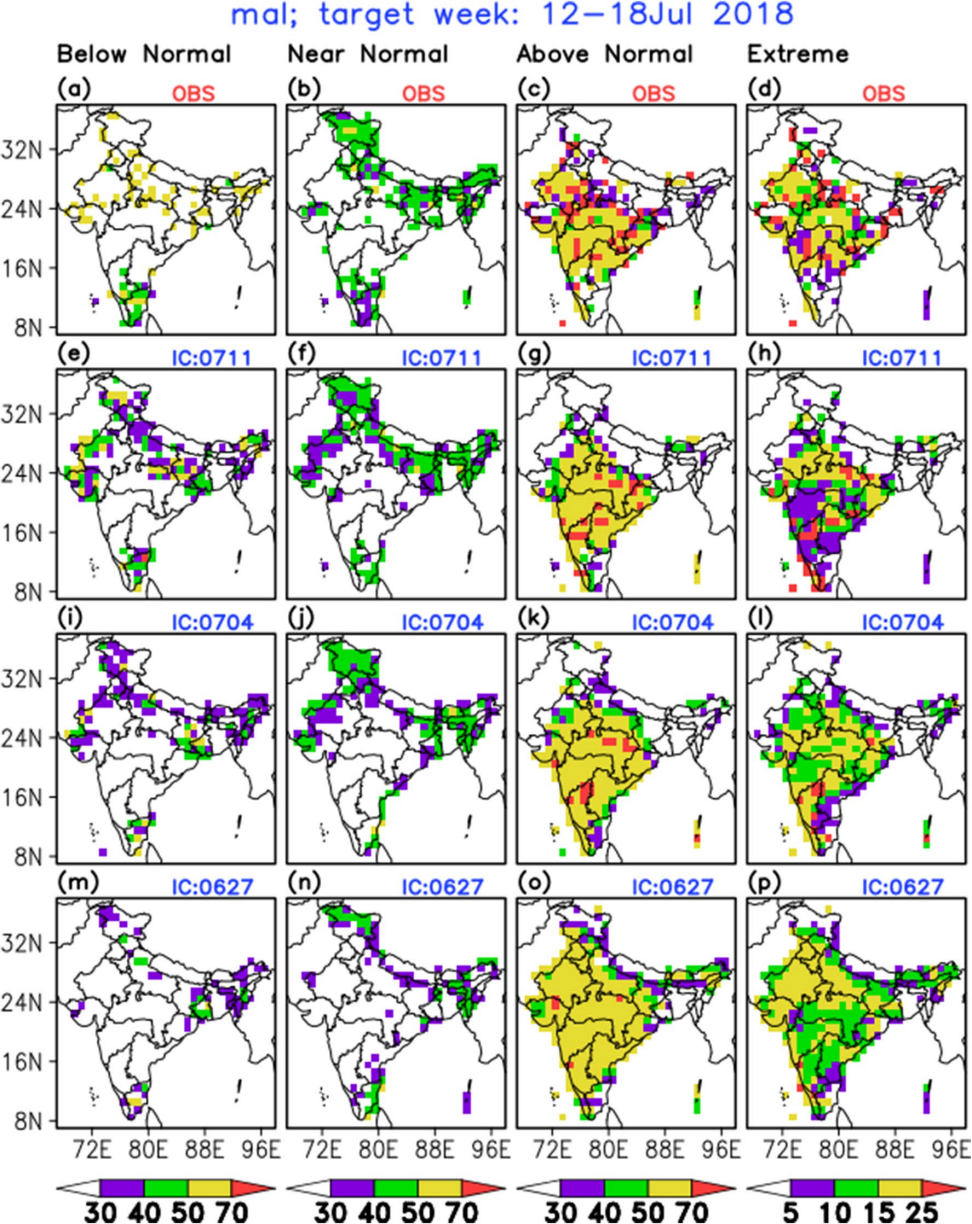
Probabilities of below normal (BN), near normal (NN), above normal (AN) and extreme (EXT) category of occurrences of MAL during 12–18 July 2018 for observation (**a**)–(**d**) and forecasts from initial conditions 11th July (**e**)–(**h**), 4th July (i)–(l) and 27th June (**m**)–(**p**) of 2018. (This map is generated using GrADS (version 2.0.2.oga.1), https://cola.gmu.edu/grads/).

The previous skill analysis and probabilistic forecast verification give the confidence to use this 6 × 6 SOM based EHWS for the real-time extended range prediction (ERP) (i.e. 2–3 weeks in advance) of the incidence of these diseases. For the real-time ERP of MAL and ADD over the whole Indian region, the gridded data ($1^\circ \times 1^\circ$ resolution) of R/F, TMx and TMn (all bias-corrected) are considered from the model, a multi-model ensemble forecast system for weather parameters (please refer Data and Methods section). Figure 5 shows the verification of the model forecast for MAL cases during the target week 12–18 July 2018. From the observed climatological weekly number of MAL/ADD cases (Fig. 1), it is seen that July experiences very high number of cases. So the target week is arbitrarily chosen during that month. Figure 5a–d show the observed probabilities of BN, NN, AN and EXT categories respectively. Here, it will be worth mentioning that the observed number of MAL cases are not the actual recorded cases over all grids, but they are produced using the observed gridded R/F, TMx and TMn datasets as inputs in the SOM technique, by assuming that this model is perfect since we don’t have the actual observed health data for all grids of the country. This analysis is done only for the verification purpose. Figure 5e–h represent the same four categorical probabilistic forecasts based on the 11th July, 2018 initial condition (IC) i.e. week-1 lead. The same forecasts from 4th July (week-2 lead) and 27th June (week-3 lead) 2018 ICs are placed in Fig. 5i–l and m–p respectively. From the Fig. 5a, it can be observed that during this target week most of the places over India (except western, eastern and northern parts), the probability of BN occurrence of MAL is less and it is reasonably predicted by the nearest two ICs (Fig. 5e and i). For the NN category, over north, eastern parts and extreme southern parts experiencing about 40% or more probability (Fig. 5b), and which is predicted reasonably from all nearest three ICs (Fig. 5f, j and n) with the decreasing probabilities from farthest IC. Most importantly for the AN cases (Fig. 5c, g, k and o), the western, central, south-east and parts of south India are having higher probability ($>50\%$ and somewhere $>70\%$), and the EHWS could predict this above normal type condition reasonably well from all the leads, though there are over predictions over parts of northern and north-eastern India from longer lead. The EXT category can be a great threat to the population. Figure 5d shows the observed pattern for MAL over the country for extreme cases, which shows that there is more than 15% chance over the north-west, central and south-eastern parts of the country and it is significantly captured by EHWS well in advance (Fig. 5h, l and p) albeit with some spatial error.

A similar verification plot for MAL in the target week 05–11 July 2018, is shown in Figure S16. For ADD and target weeks 05–11 and 12–18 July 2018 the verification plots are provided as Figure S17 and S18 respectively. By analysing these three additional verification plots, it is observed that the newly developed EHWS is able to provide the chances (% probabilities of BN, NN, AN and EXT) of occurrences of MAL and ADD over different places of India reasonably well in advance that is at least 2 weeks in advance.

## Discussion and conclusions

Changes in weather patterns along with the extreme weather events can affect the transmission of infectious diseases, besides population dynamics, disease control strategies, sanitation, human activities such as deforestation, irrigation, swamp drainage etc. Children (particularly from urban slums in India) who belong to poor socioeconomic status had a higher ADD incidence than the better socioeconomic group^32^. A report by UNICEF^33^ stated that the poor sanitation, lack of access to clean water, and inadequate personal hygiene are responsible for an estimated 88% of childhood ADD in India.

We conducted a proof of concept analysis that an EHWS can be developed utilizing climate forecasts. This is one of the mandates of India Meteorological Department under WMO Global Framework for Climate Services. This can be used by India meteorological Department as a tool for Climate Services towards health sector. Thus, we develop an EHWS for the real-time prediction of disease incidences over the Indian region by using the weather parameters (R/F, TMx and TMn) as predictors. Since the thresholds/ranges of different weather parameters for the occurrence of such diseases are region-dependent (as seen in the case of PNE and NGP), the thresholds/ranges that are valid for one region can give wrong information for other regions. Therefore, there should be a unique technique that can inherently take care of this issue. For this, a pattern recognition technique viz. SOM, which uses the normalized climatic variables as input variables, is used as a prediction tool for such disease incidences. Two separate SOM analyses (3 × 3 and 6 × 6) have been performed to obtain the reference vectors for individual nodes by using the gridded weather parameters for all grids over the Indian subcontinent. Here, the effect of monthly and fortnightly prevailing weather conditions are considered by incorporating the 1 W, 2 W and 4 W mean values. Using these reference vectors and the weather parameters, two separate clusterings (3 × 3 and 6 × 6) have been done for PNE and NGP districts separately (please refer to the Data and Methods section). Then, the mean maps and class-wise probabilistic maps of the individual node from 3 × 3 SOM analysis (for simple visualization) are obtained to figure out the relationship between meteorological parameters and disease incidence for both the regions. It is clear from the results that the increased probabilities of high (less) R/F, high (low) TMn and low (moderate) TMx are more (less) conducive for MAL and ADD over both regions, but with different thresholds/ranges. Based on the skill analysis in terms of CC, RMSE and BSS, it is found that the EHWS based on the 6 × 6 SOM has significant skill as compared to the climatological forecasts (please refer Table 1). The deterministic as well as the categorical probabilistic forecasts are obtained during the period 2009–2016 and climatological biases have been removed and then compared with the observed climatological values. It is noted from the deterministic forecast that, even though this forecasting system could capture the variability of the disease incidences during different seasons over both the regions reasonably well, it failed to predict the actual number of cases for few occasions. Nevertheless, the probabilistic forecasts for different categories such as BN, NN, AN and EXT are found to be more useful and practical than the deterministic ones and can provide more scientific information than the climatological forecasts.

This provides the optimism that the SOM based forecasting system (based on 6 × 6 clustering) for disease incidences (in this study we checked with ADD and MAL over PNE and NGP) has great potential in capturing the variability during different seasons and can give useful realistic and scientific information for the policymakers about the potential incidence of these diseases by clustering only the meteorological parameters. It indeed gives the confidence to use this method for the real-time prediction of such disease incidences over the whole country based on the available gridded weather parameters. Using this information, we made the real-time prediction of such events over each grid points in India to provide an outlook for these diseases in terms of probabilities of BN, NN, AN and EXT categories reasonably well in advance (i.e. at least 2 weeks in advance). We hope that this type of information obtained from the EHWS may benefit decision-makers through the advanced and targeted allocation of resources for combatting disease epidemics in India. The possible interventions when there is a forecast of a high probability of ADD/MAL incidences could be: (i) For ADD: the management of safe drinking water from the source to tap or in individual house and proper water storage management for safe drinking water, making sure the adequate sanitation for individual household or at the area of possible outbreaks, washing hands with soap at critical times (e.g. before taking of food and after toilet use), improvement of hygiene and also, the campaign to create community health awareness is required; and (ii) For MAL: timely diagnosis and treatment, distribution of protective insecticidal bed nets, effective surveillance and reporting, and health education campaigns.

### Limitations and alternatives

While socioeconomic factors, access to appropriate hygiene and clean drinking water are also important factors for transmission of ADD, MAL and other diseases, this study focuses on the association between the variability in the meteorological variables and disease incidence. In the present study, we have not incorporated all these above information as inputs to the EHWS due to the non-availability of the information. We agree that the inclusion of these parameters is important and omitting such influences may lead to either variation in disease incidence being incorrectly attributed to climate effects and/or poor predictive accuracy.

Although the role of the meteorological parameters in the transmission of MAL and ADD diseases are established only based on R/F, TMx and TMn, a detailed skill analysis is performed by incorporating the relative humidity (RH) parameter along with the other three, but the reference vectors, in this case, are calculated taking PNE and NGP regions together (as we do not have a good quality historical as well as real-time gridded humidity datasets over Indian region). It is found that there are no significant changes in the skill scores with the inclusion of RH (please refer to Table-S1 in Supplementary Information). It has to be noted that daily data in real-time is only available for R/F, TMx, TMn at India Meteorological Department (IMD) website. Therefore, the whole analysis is done using only three meteorological parameters as inputs. It is to be noted that the datasets available for MAL and ADD in this study are only for the period 2012–2016 and only for two places.

We believe that the model can perform much better if we can incorporate more health data covering more years and regions. This EHWS can be applied to other diseases also, which are influenced by the different weather parameters. Therefore this study proposes a more strong collaboration between the meteorological and health department for data sharing of more diseases, more regions and in longer time scales. Linking health and environmental information across high spatial and temporal resolutions using historical and real-time surveillance information can inform the development of these early warning systems to save lives.

## Data and methods

In this study, the meteorological variables (gridded as well as for the different stations), weekly mean maximum temperature (TMx), weekly mean minimum temperature (TMn) and weekly total rainfall (R/F) are obtained from the archival of IMD. The health data for acute diarrheal diseases (ADD) and malaria (MAL) for two districts, Pune (PNE) and Nagpur (NGP) in the state of Maharashtra, India have been obtained from the Directorate of Health, Govt. of Maharashtra. The above datasets have been used to find out the relationship between disease incidences and meteorological variables, using Self Organising Map (SOM)^28,29^. The weekly data of meteorological variables over the period 2009–2016 have been used to calculate 1 W (only current week), 2 W (considering current week and previous week) and 4 W (considering current week and last three weeks) weekly averaged values (TMx and TMn) and weekly accumulated values (R/F) for each week during a calendar year. This is done to include the effect of monthly and fortnightly prevailing climatic conditions. The weekly total ADD and MAL cases for PNE (NGP) are available for the period 2012–2016 (2012–2016) and 2013–2016 (2012–2016) respectively. We have also considered the population factors for both the regions. The population datasets are available over these locations with 10 years interval. However, one approximation has been made while converting them as weekly population by considering the linear regression/interpolation method. Since the weekly number of MAL cases are very less compared to ADD cases for both the regions, just to rescale them the MAL cases are considered as per 10 million populations and ADD cases are considered as per 1,00,000 populations.

SOM is basically a pattern recognition technique based on unsupervised learning neural networks (i.e. without prior knowledge of the data domain). Giving an N-dimensional data space (i.e. input variables), the SOM algorithm distributes an arbitrary number of nodes in the form of a 1-D or 2-D regular lattice. Each node is uniquely defined by a reference vector consisting of weighing coefficient and therefore different weather variables are normalized before using in SOM as they have very different mean and distribution. The adaptation of the reference vector in accordance with the input vector is done through the minimization of Euclidean distance between the reference vector for any particular node and the input data vector. Two separate SOM clustering have been done with (i) 3 × 3 lattice (total 9 nodes) and (ii) 6 × 6 lattice (total 36 nodes). The 3 × 3 clustering is used for easy visualization of the capabilities of the SOM method while the 6 × 6 clustering is used for real forecasting. All the 9 weather parameters (4 W, 2 W, 1 W values of R/F, TMx and TMn), for each grid and two stations (PNE and NGP), are standardized before using them as input to SOM. The standardization (see Eq. 1) has been done to remove any local variation that arises due to the variables having a different mean and distribution across the grids/regions.

1 $${X}_{std}=\frac{{X}_{actual}-{X}_{min}}{{X}_{max}-{X}_{min}}$$

where ${X}_{std}$ is the standardized value, ${X}_{actual}$ is the actual value, ${X}_{min}$ and ${X}_{max}$ are the 5th and 95th percentile values of the respective variables for individual grid/region.

At first, the reference vectors for individual nodes are obtained by clustering only weather parameters and by considering all grids (~ 350 grid points) over the Indian region. To obtain these, 9 weather parameters over the period 1998–2013 (total data points $\sim 350\times 16\times 52\times 9$) are trained using the SOM algorithm. This is done for 3 × 3 and 6 × 6 clustering separately. Now, using this reference vectors and 9 normalized weather variables over the period 2009–2016 (total data points: 8 × 52x9) as inputs in SOM, two separate clustering (3 × 3 and 6 × 6) have been performed for each location, PNE and NGP separately. Thus, it will group them in different nodes according to the best match, with the help of reference vectors. So, we have 9 (from 3 × 3 SOM) and 36 (from 6 × 6 SOM) groups of weather variables which will represent 9 and 36 different relationships of weekly weather variables. Then weekly ADD and MAL cases, corresponding to those weeks (from weather variables) which are clustered in different nodes, are also grouped in different nodes. Here, the disease datasets are from 2012 to 2016, but the weather variables are clustered over the period 2009–2016. So for disease events in each node there will be many missing values (which are kept as undefined values). So, in this way we have different weekly weather and disease events in different nodes for two sets (3 × 3 and 6 × 6) of clustering for individual regions. The idea behind this is to see the usefulness of this technique in the health sector, i.e. to examine whether disease incidences can be foreseen using the weather variables (considering current, fortnightly and monthly weather condition) over different locations. Now, in each node, there will be a distribution of the (i) R/F, TMx and TMn with 1 W, 2 W and 4 W time steps and (ii) ADD and MAL with 1 W time step only. From the clustered datasets in each node, we can have different statistical parameters like mean, median, quartile values, percentile values etc. The mean maps of R/F, TMx, TMn, ADD and MAL for both regions are obtained from 3 × 3 SOM analyses for easy visualization of the relationships between health and weather variables. The actual distributions of these 9 weather parameters and 2 diseases around the mean in different nodes are obtained from 3 × 3 SOM clustering and presented through the Box-and-Whisker diagrams^30^ for both the regions (placed in Supplementary Information as Figure S3 and S4). A boxplot is a standardized way of displaying the distribution of datasets based on some statistical parameters (median, first quartile, third quartile and outliers). It gives an idea about the datasets whether it is symmetrical and how tightly data is grouped, or it is skewed.

Similarly, to obtain the forecasted value for a particular week of a particular year, total 9 weather parameters are used as inputs in 6 × 6 SOM clustering. It will cluster them as per the reference vectors (which were obtained considering all grids) and will group them in a particular node. From the previous step, we have mean values and different statistical threshold values for different nodes. So, the mean value of that particular node will be the output as the deterministic forecast for that particular week. Likewise, the weekly deterministic forecasted values of MAL and ADD over two regions are calculated separately for every 52 weeks of each calendar year during the observation period (2009–2016) using only the weather variables. Besides, the categorical probabilistic forecasts for every 52 weeks of each year during 2009–2016 are obtained using the tercile and percentile values of ADD/MAL in each node instead of mean values. The categorical forecasts, below normal (BN), near normal (NN), above normal (AN) and extreme (EXT) are considered when they satisfy the conditions (i) $<Q1,$(ii) $\ge Q1 and \le Q3,$(iii) $>Q3 and \le p95$ and (iv) $>p95$ respectively where, Q1 is lower tercile, Q3 is upper tercile and p95 is the 95th percentile.

To evaluate the skill of this EHWS, the Correlation Coefficient (CC) and Root Mean Square Error (RMSE) are calculated using the weekly actual observed ADD/MAL cases and deterministic bias-corrected forecasts (see Eq. 2) during 2009–2016. Total 208 and 260 data points (data point represents the total number of weeks during the study period) for MAL are considered for PNE and NGP respectively; whereas, total 260 data points for each region are taken into account for ADD. Subsequently, the Brier Skill Scores (BSS)^31^ for different categorical probabilistic forecasts (BN, NN and AN (here it is considered as $>Q3$, so it will include the EXT as well)) are calculated concerning the actual observations over these two regions separately and over the period 2009–2016.

2 $${F}_{bc}\left(i,j\right)={F}_{raw}\left(i,j\right)-{F}_{clim}\left(j\right)+{O}_{clim}\left(j\right)$$

where, ${F}_{bc}, {F}_{raw}$, ${F}_{clim}, {O}_{clim}, i and j$ represent the bias-corrected forecast, raw forecast, weekly model climatology, weekly observed climatology, year and week of a calendar year respectively.

For the real-time Extended Range Prediction (ERP) of such disease incidences using the 6 × 6 SOM based EHWS, the model gridded data for weather parameters are used from a multi-model ensemble (MME) prediction system^34–38^ (based on Climate Forecast System version 2), indigenously developed at Indian Institute of Tropical Meteorology (IITM), India and operationally run at IMD, India. This MME forecasting model generates real-time forecasts for different weather parameters on a weekly basis^39^, which is significantly skilful up to 2–3 week lead time (thereafter they will be represented as week-1 lead i.e. 1–7 days average, week-2 lead i.e. 8–14 days average and week-3 lead i.e. 15–21 days average) in predicting rainfall/temperatures during the relevant seasons and hence can be used for operational purposes^40^. The model data of R/F, TMx and TMn are used after removing the climatological bias with respect to observation^39^. So, after developing the suitable relationship between different weather parameters and the disease incidences through the SOM technique, we assess the potential applicability of real-time monitoring and prediction of incidence probabilities of such diseases with a lead time of 2–3 weeks to inform public health actions. The real-time prediction of disease incidences (here ADD and MAL) have been generated using the bias-corrected weekly forecasts of R/F, TMx and TMn for 4 weeks lead time. For 4-weeks lead forecast of disease incidences, the 9 weather parameters are calculated using the available forecasted weekly values and previous week’s observed values (before the forecast date, since model data can be available) and clustered using 6 × 6 SOM. For the week-1 forecast of disease incidences, the 9 weather parameters are clustered and they are calculated as: (i) 1 W: considering week-1 forecasted values, (ii) 2 W: considering week-1 forecast and previous week (from the model initialization date) observation and (iii) 4 W: considering week-1 forecast and previous three weeks observed values. Likewise for week-2 forecast of disease incidences, 9 weather parameters are calculated as: (i) 1 W: considering week-2 forecasted values, (ii) 2 W: considering week-2 and week-1 forecasts and (iii) 4 W: considering week-2, week-1 forecasts and previous two weeks observed values. Similarly the week-3 and week-4 forecasts of different disease incidences are obtained. In this study the probabilistic forecasts for ADD and MAL are obtained in similar way, discussed in the paragraph before last, but combining the nodes from two regions to calculate different statistical thresholds for ADD and MAL in each clustered node (total of 36 nodes).

For the verification of real-time forecasts for all grids, the observed weekly categorical probabilistic forecasts of MAL and ADD are generated by using the gridded observation datasets of R/F^41^, TMx and TMn^42^ as inputs in the 6 × 6 SOM technique, since the actual observed cases for MAL and ADD are not available for all other grids/regions (here we assume that the SOM based forecasting system is perfect). In this study, we have shown the model verification up to week-3 lead. The whole process of SOM clustering and forecasting strategy can be visualised from Figure S1 in the Supplementary Information.

## Supplementary information

Supplementary Information

Supplementary Data

## Data Availability

All weather and health datasets for two study locations, gridded observation and model datasets from different initial conditions used in this study are provided as Supplementary_data.zip. Since the size of the raw model datasets (with different models, different ensembles etc.), used in this study to prepare the final products is too big, it is difficult to attach them as supplementary datasets. Therefore, they will be made available in the following link https://www.tropmet.res.in/monsoon/monsoon2/ (upon request). The other products generated during this study are available from the corresponding author upon reasonable request. The health data used in this study was provided by one of the co-authors of this study and it is not publicly available and the permission was obtained from the appropriate authority to use this data for research purpose.

## Code availability

Analytical scripts and programming used in this study are available from the corresponding author upon reasonable request.
