# Supplementary material for: Development of a probabilistic early health warning system based on meteorological parameters
Source: Sci Rep. 2020 Sep 8;10:14741. doi: 10.1038/s41598-020-71668-6 (PMC7479102; doi:10.1038/s41598-020-71668-6)
Supplement: Supplementary file 1 — Supplementary Information [file 41598_2020_71668_MOESM1_ESM.pdf]

Development of a probabilistic early health warning system based on meteorological parameters

A. K. Sahai[1*], Raju Mandal[1,2], Susmitha Joseph[1], Shubhayu Saha[3], Pradip Awate[4], Somenath Dutta[5], Avijit Dey[1], Rajib Chattopadhyay[1], R. Phani[1], D. R. Pattanaik[6] and Sunil Despande[5]

[1] Indian Institute of Tropical Meteorology (IITM), Pune, India

[2] Department of Atmospheric and Space Sciences, Savitribai Phule Pune University, India

[3] Rollins School of Public Health, Emory University, GA, USA

[4] Integrated Disease Surveillance Program, Maharashtra, India

[5] India Meteorological Department (IMD), Pune, India

[6] India Meteorological Department (IMD), New Delhi, India

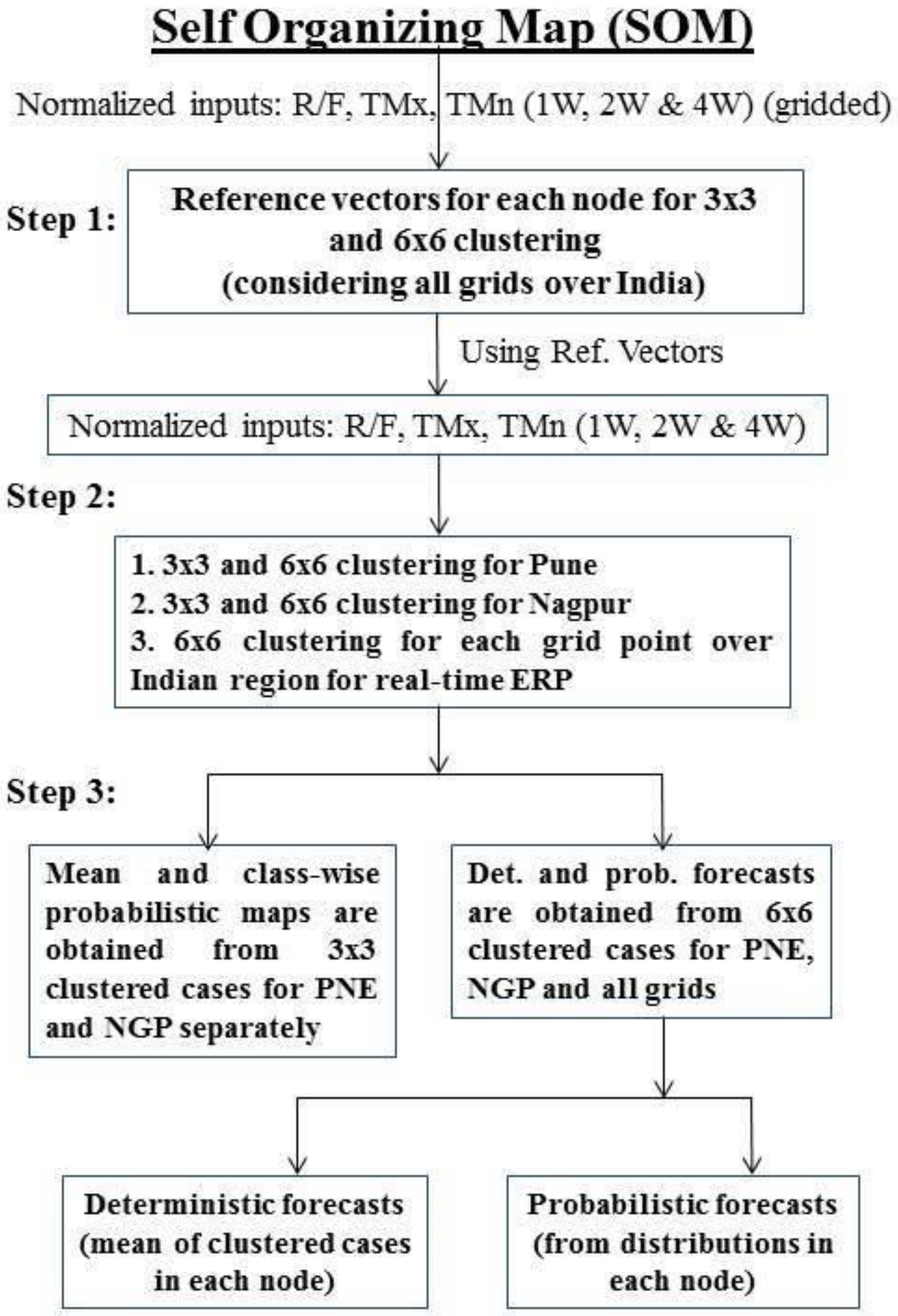

Figure S1: Self Organizing Map analysis technique followed in this study.

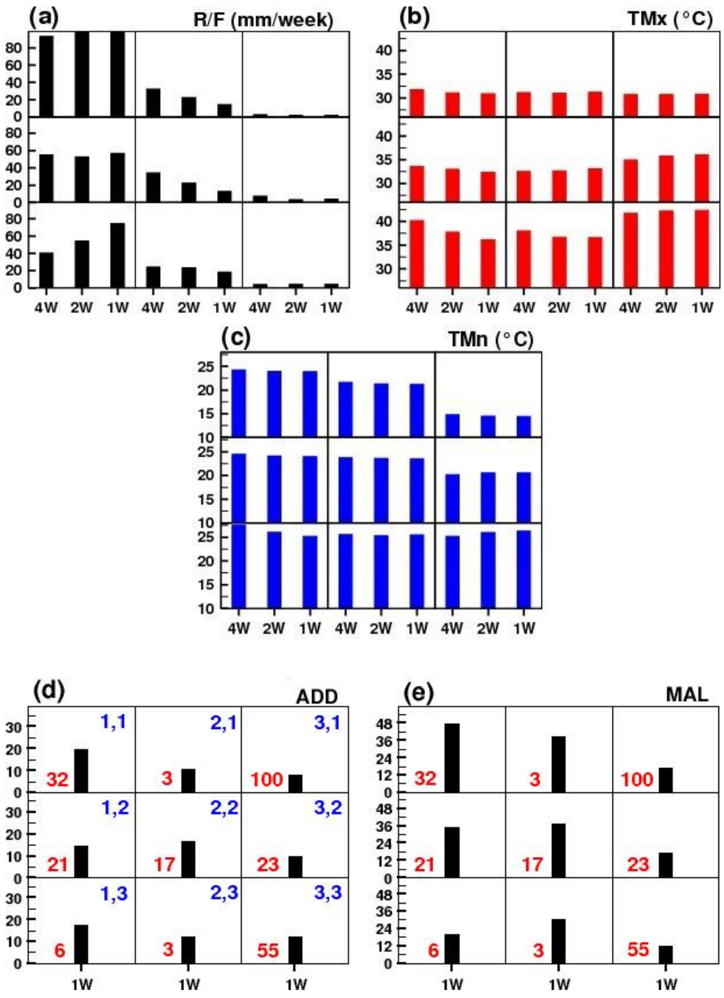

Figure S2: Mean map of all the cases clustered in each of 9 nodes obtained from 3x3 SOM clustering for Nagpur: (a) R/F, (b) TMx, (c) TMn and (d) ADD cases (per 1,00,000 populations) and (e) MAL cases (per 10 million populations). Here, the positions of the nodes are mentioned on right-top of the each sub-panel (Fig.S2d) and the numbers of cases are mentioned in red colour for ADD and MAL (Fig.S2d and S2e).

Over Nagpur, it is observed that, the highest valued ADD and MAL cases are clustered in nodes (1,1), while the minimum valued cases are clustered in node (3,1) and node (3,3) for ADD and MAL respectively (Fig.S2d-e). The highest number of ADD and MAL cases are associated with the heavy R/F activities in all 4 weeks (range: 93-104 mm/week), high TMn $(\sim 24℃)$ and moderate TMx values $(range: 31 - 32℃)$ in all 4 weeks. The lowest ADD cases (node (3,1)) are observed to link with no rain, low TMn $(\sim 15℃)$ and moderate TMx $(\sim 31℃)$. Whereas the lowest cases of MAL (node (3,3)) is linked with no rain, TMx is very high $(\sim 42℃)$ and Tmn is also considerably high $(\sim 25 - 26℃)$. In conclusion, the higher number of ADD and MAL cases are grossly associated with (i) large or moderate amount of rainfall activities, (ii) high values of TMn and (iii) moderate TMx values during all 1W, 2W and 4W weeks. Again the less number of ADD and MAL cases are associated to the no rain, low to moderate TMn and moderate to high TMx.

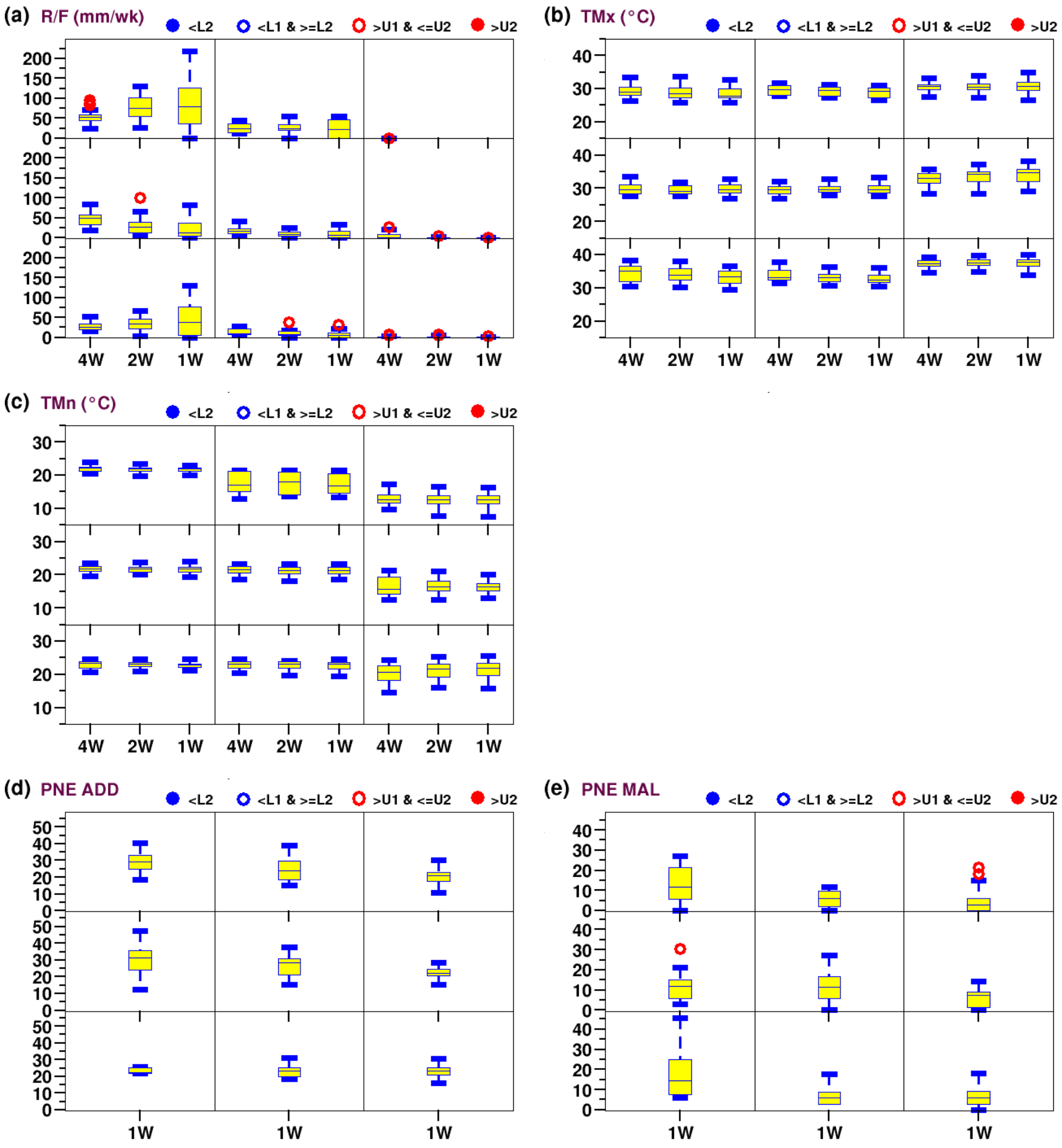

Figure S3: Box-and-Whisker diagrams for (a) R/F, (b) TMx, (c) TMn (d) ADD cases and (e) MAL cases, clustered into 9 nodes for **Pune**. [Where, U2=q3+3.0*IQR, U1=q3+1.5*IQR, L1=q1-1.5*IQR, L2=q1-3*IQR, q1=1st quartile, q3=3rd quartile, IQR=q3-q1=inter-quartile range, U2 & L2 are called Outliers and U1 & L1 are called Suspected outliers on the appropriate sides]

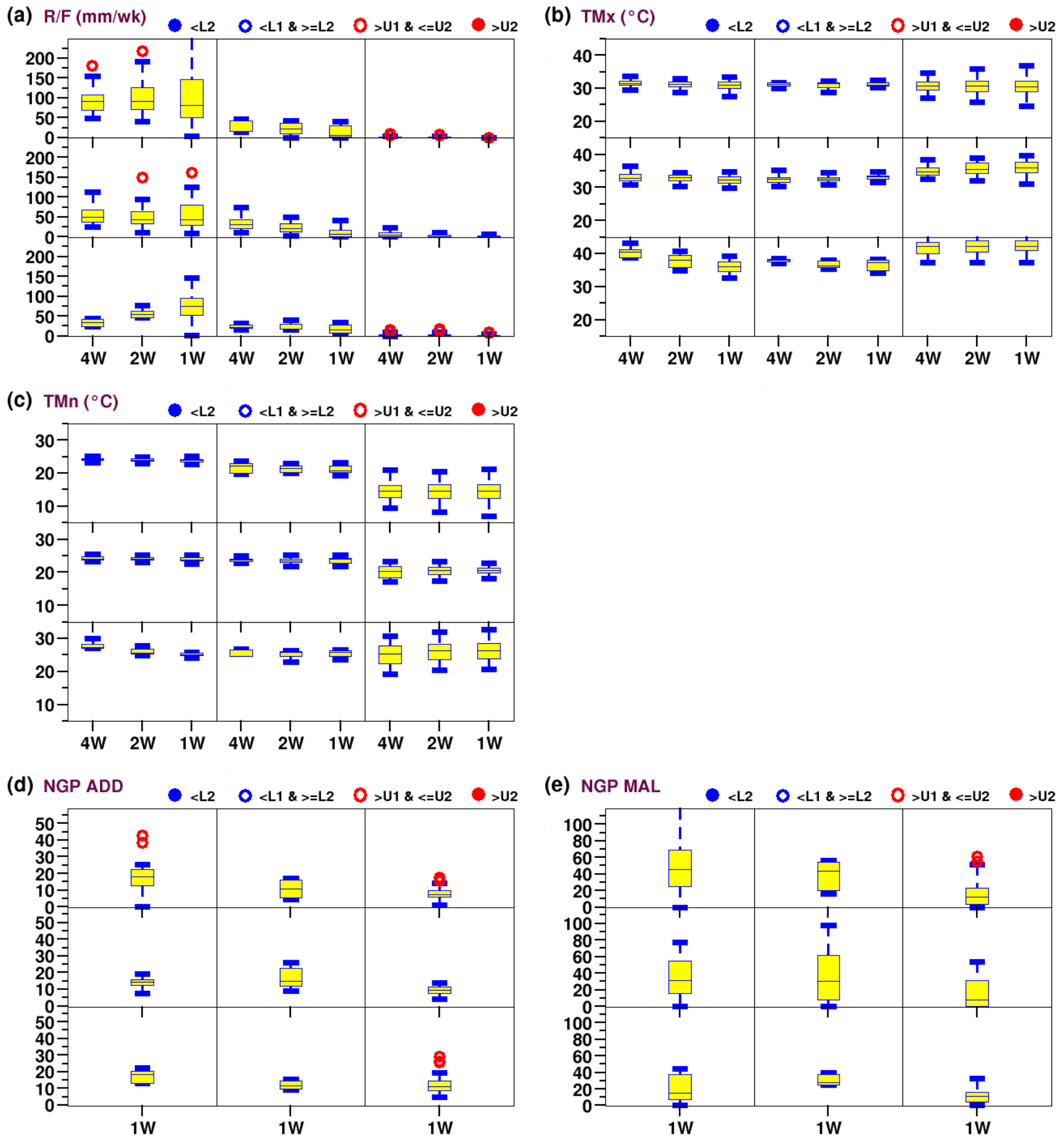

Figure S4: Box-and-Whisker diagrams for (a) R/F, (b) TMx, (c) TMn (d) ADD cases and (e) MAL cases, clustered into 9 nodes for **Nagpur**. [Where, U2=q3+3.0*IQR, U1=q3+1.5*IQR, L1=q1-1.5*IQR, L2=q1-3*IQR, q1=1st quartile, q3=3rd quartile, IQR=q3-q1=inter-quartile range, U2 & L2 are called Outliers and U1 & L1 are called Suspected outliers on the appropriate sides]

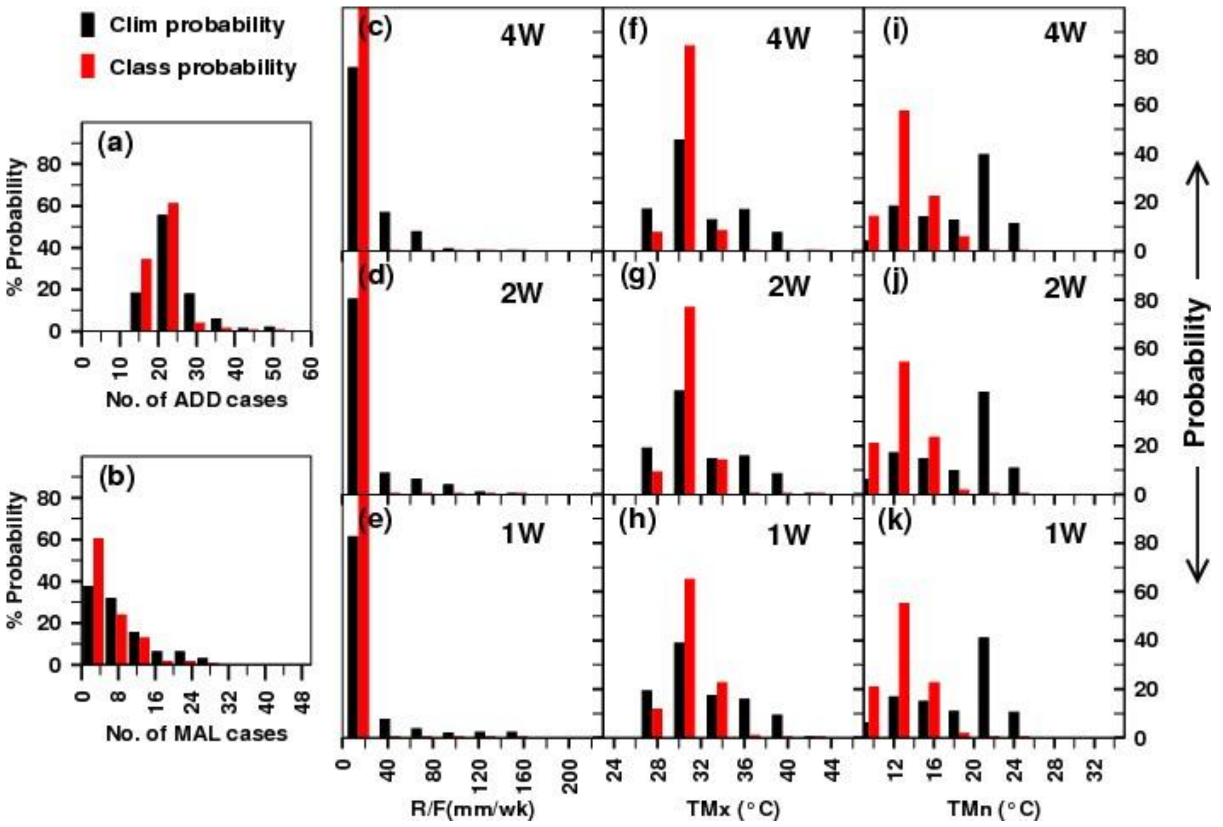

Figure S5: Class probabilities (red bars) with climatological probabilities (black bars) of: (a) ADD; (b) MAL; (c-e) R/F for 1w, 2w and 4w time steps; (f-h) TMx for 1w, 2w and 4w time steps and (i-k) TMn for 1w, 2w and 4w time steps for **node (3,1)** and for **Pune**.

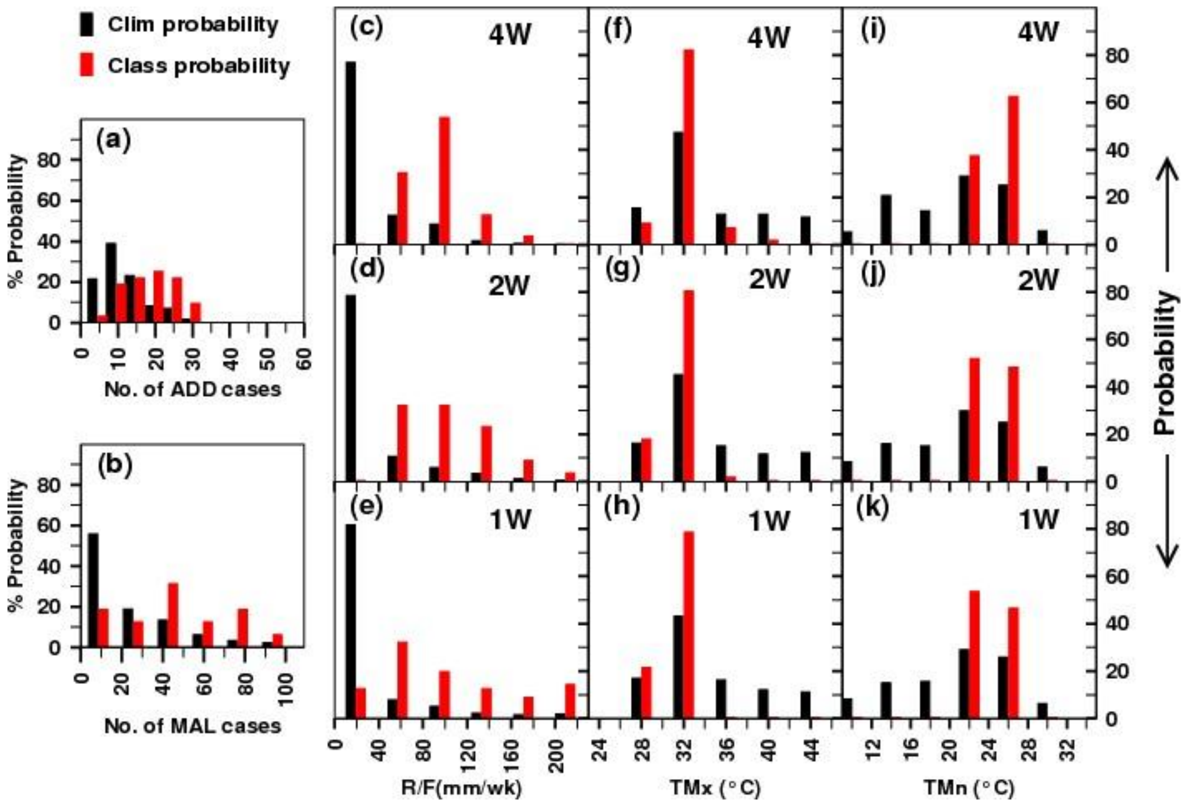

Figure S6: Same as Figure S5, but for node (1,1) and for **Nagpur**.

From Fig.S6, it is found that the increased probability of wet spell, increased probability of high TMn and increased probability of moderate TMx are more conducive for large number of ADD and MAL cases over Nagpur.

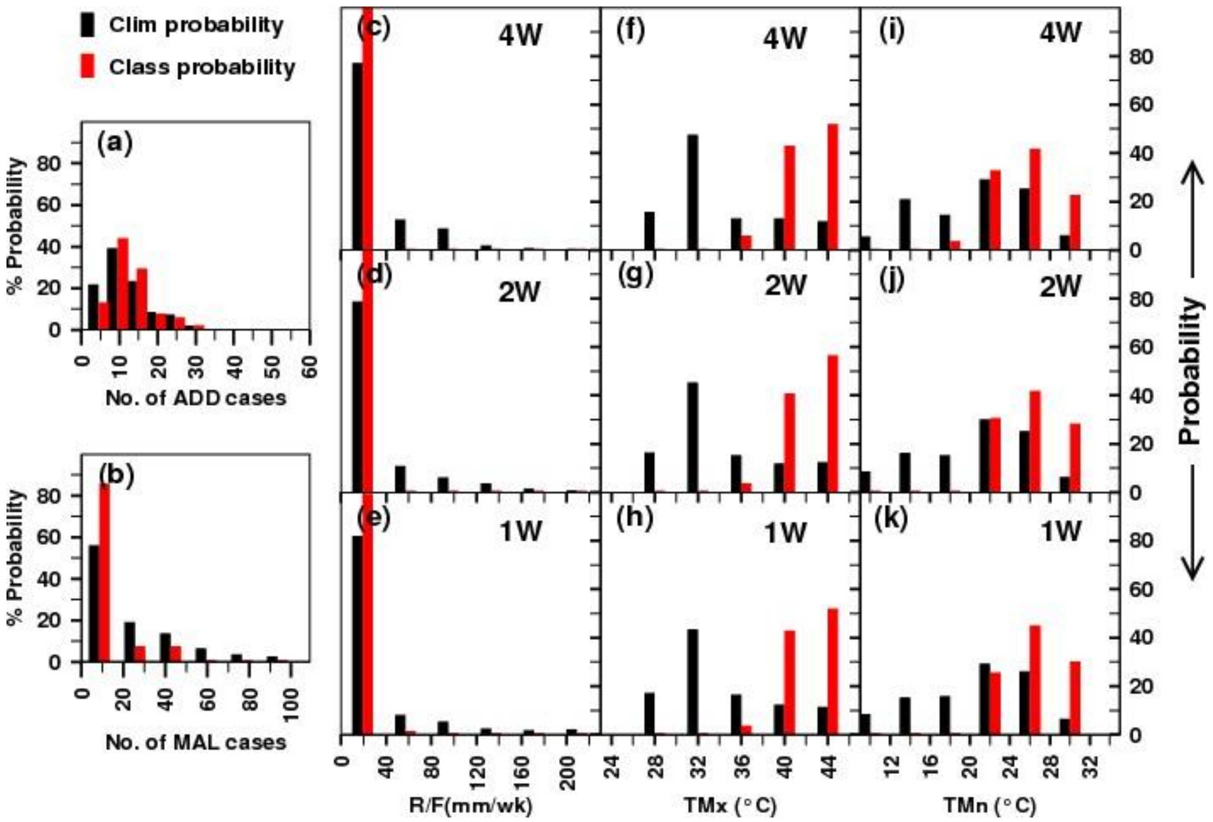

Figure S7: Same as Figure S5, but for node (3,3) and for **Nagpur**.

From Fig.S7, it is observed that, the increased probability of dry spell, increased probability of low to moderate TMn and increased probability of moderate to high TMx are less conducive for ADD and MAL over Nagpur.

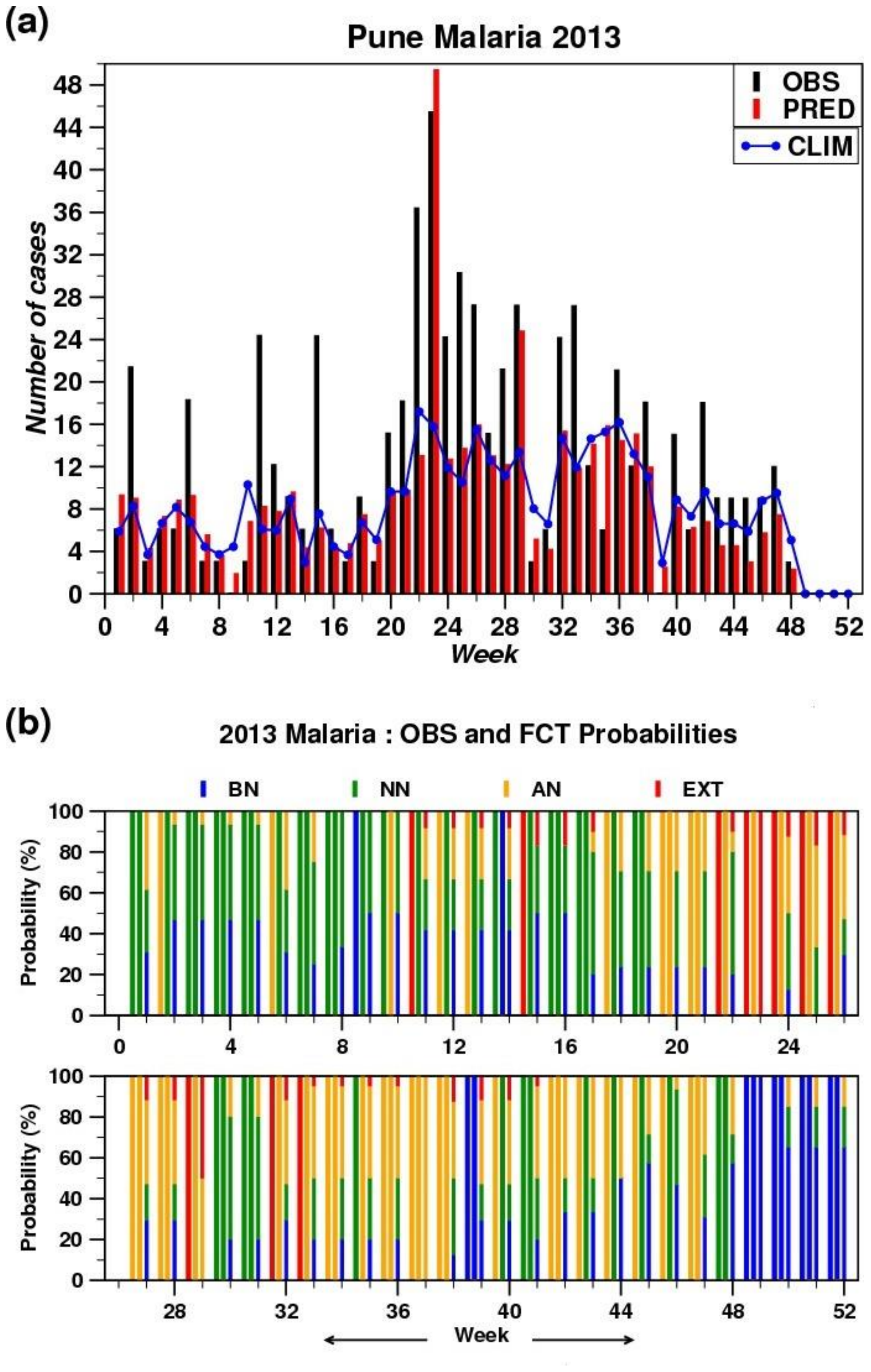

Figure S8: (a) Weekly observed values (black bars) and predicted values (red bars) and (b) probabilistic forecast: % probabilities of Below Normal (BN), Near Normal (NN), Above Normal (AN) and Extreme (EXT) occurrences for MAL during 2013 over Pune. For observation any of the above probabilities is 100%.

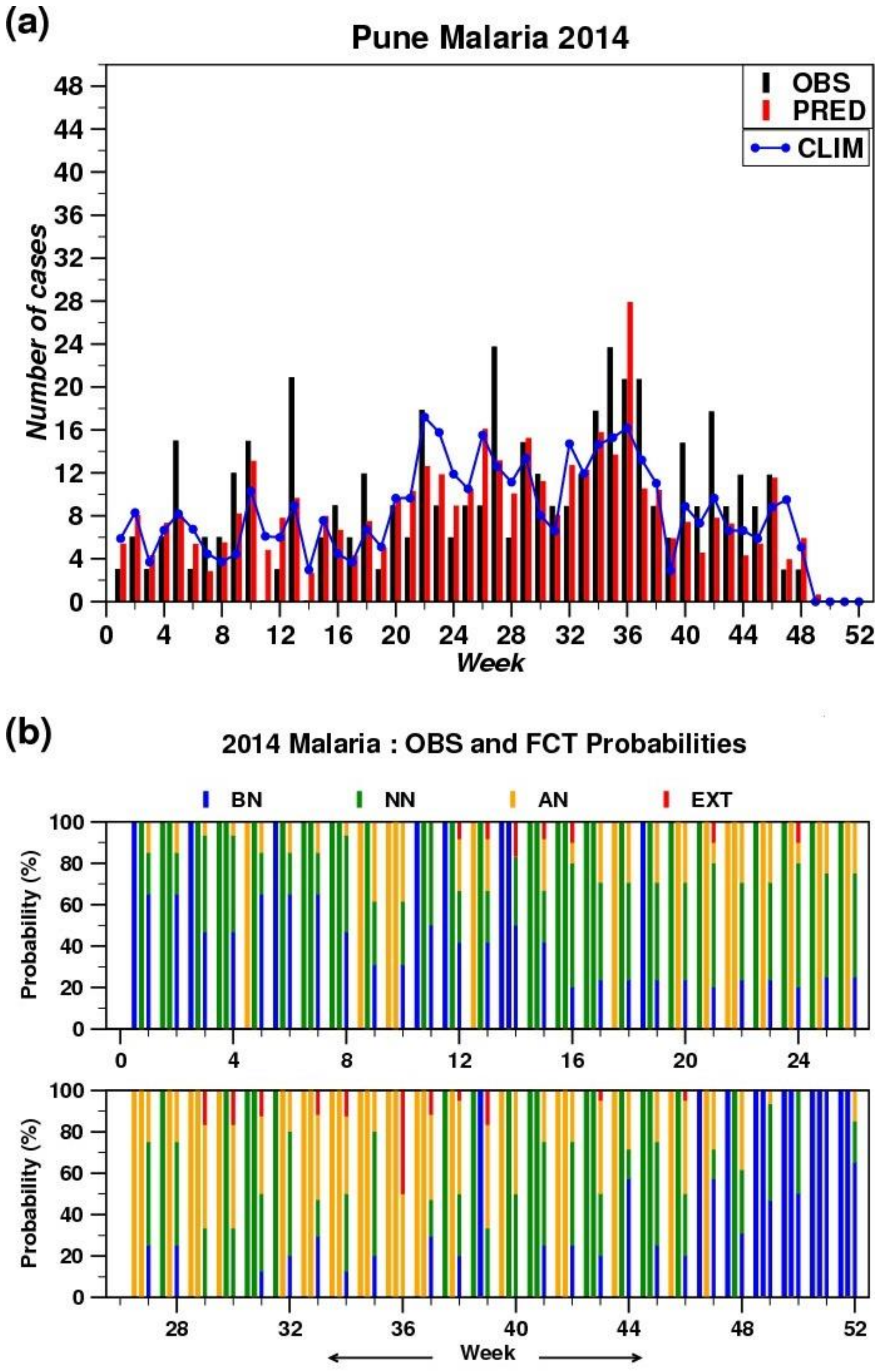

Figure S9: Same as Figure S8, but for MAL during 2014 over Pune.

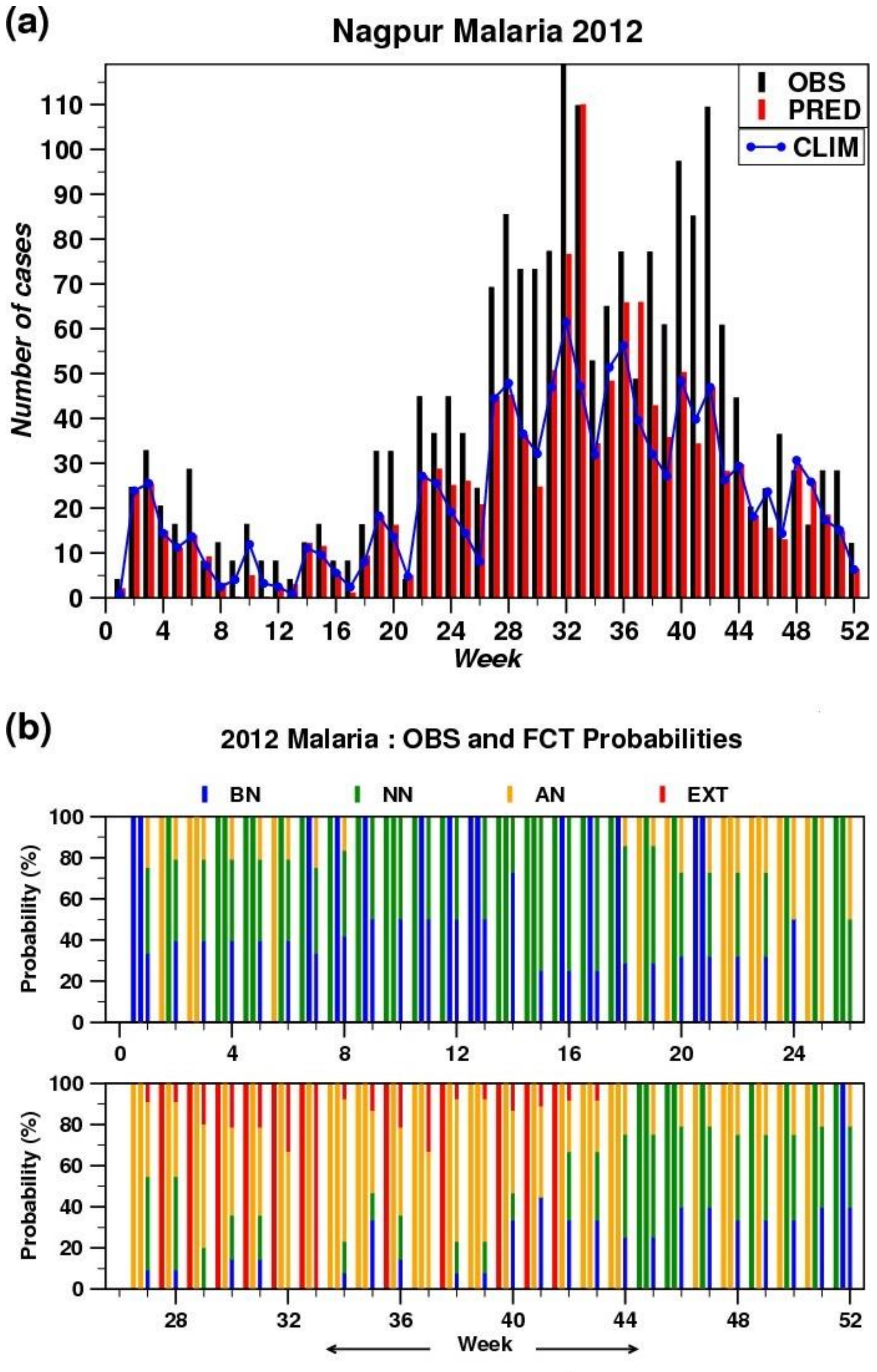

Figure S10: Same as Figure S8, but for MAL during 2012 over Nagpur.

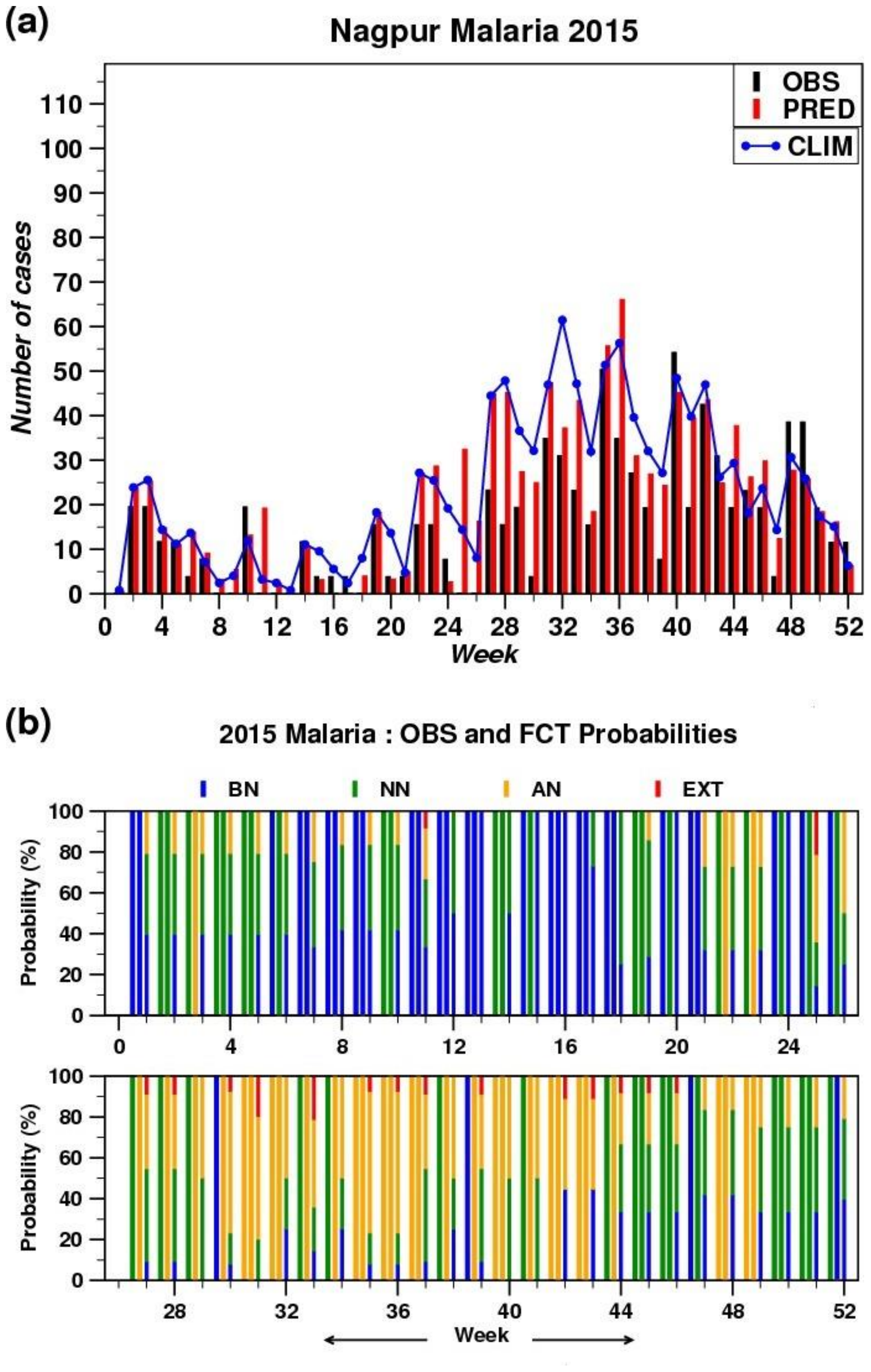

Figure S11: Same as Figure S8, but for MAL during 2015 over Nagpur.

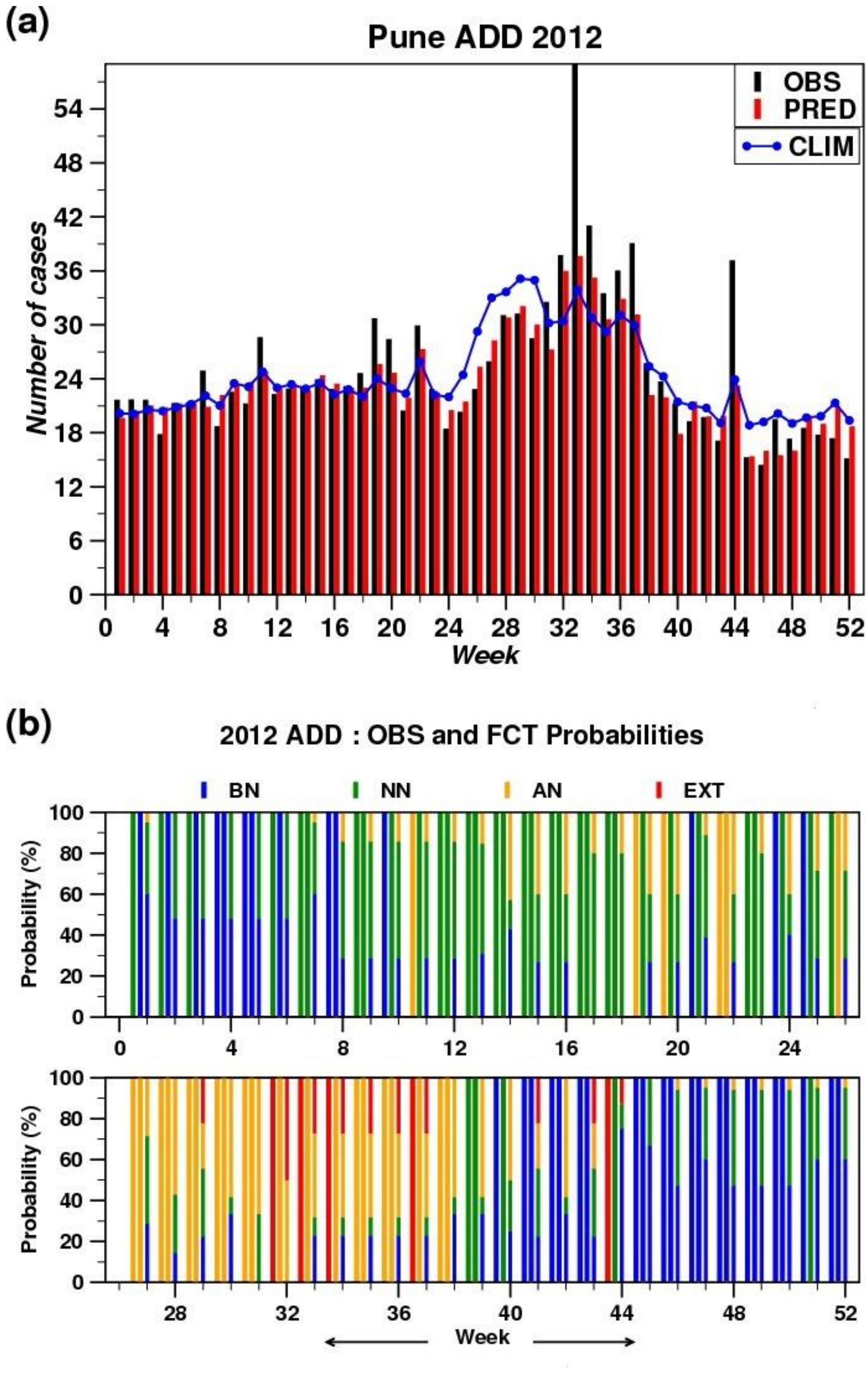

Figure S12: Same as Figure S8, but for ADD during 2012 over Pune.

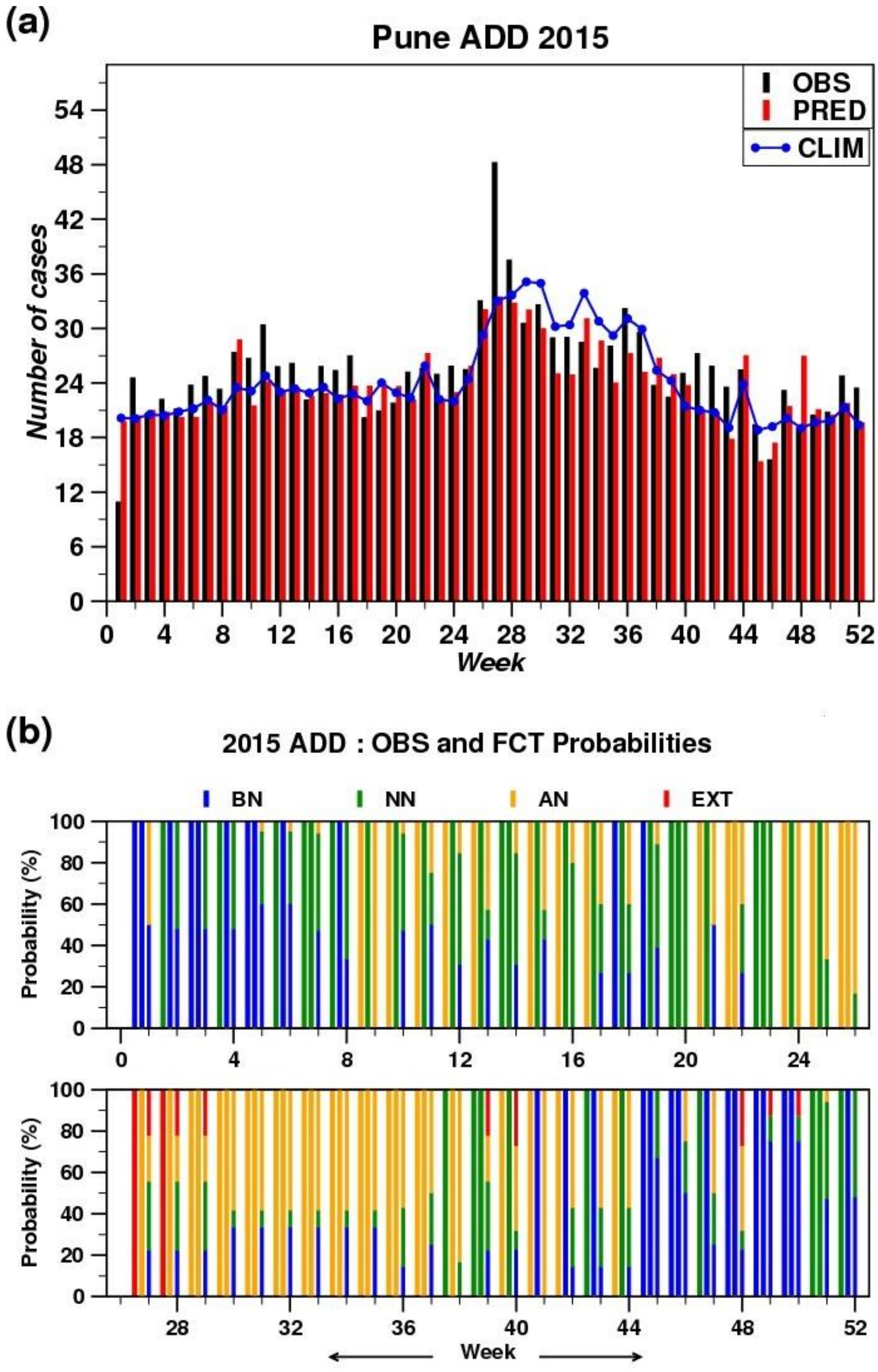

Figure S13: Same as Figure S8, but for ADD during 2015 over Pune.

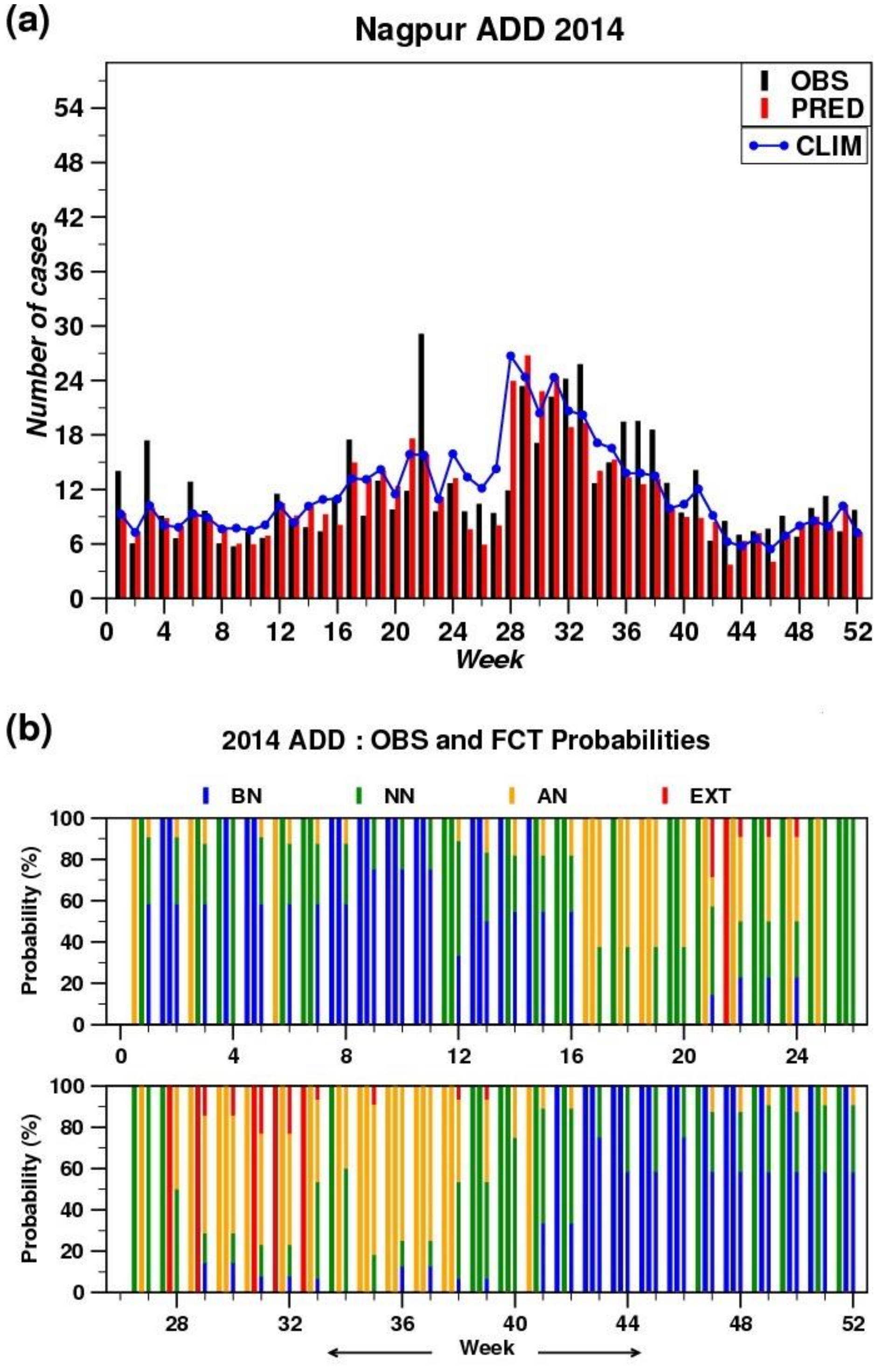

Figure S14: Same as Figure S8, but for ADD during 2014 over Nagpur.

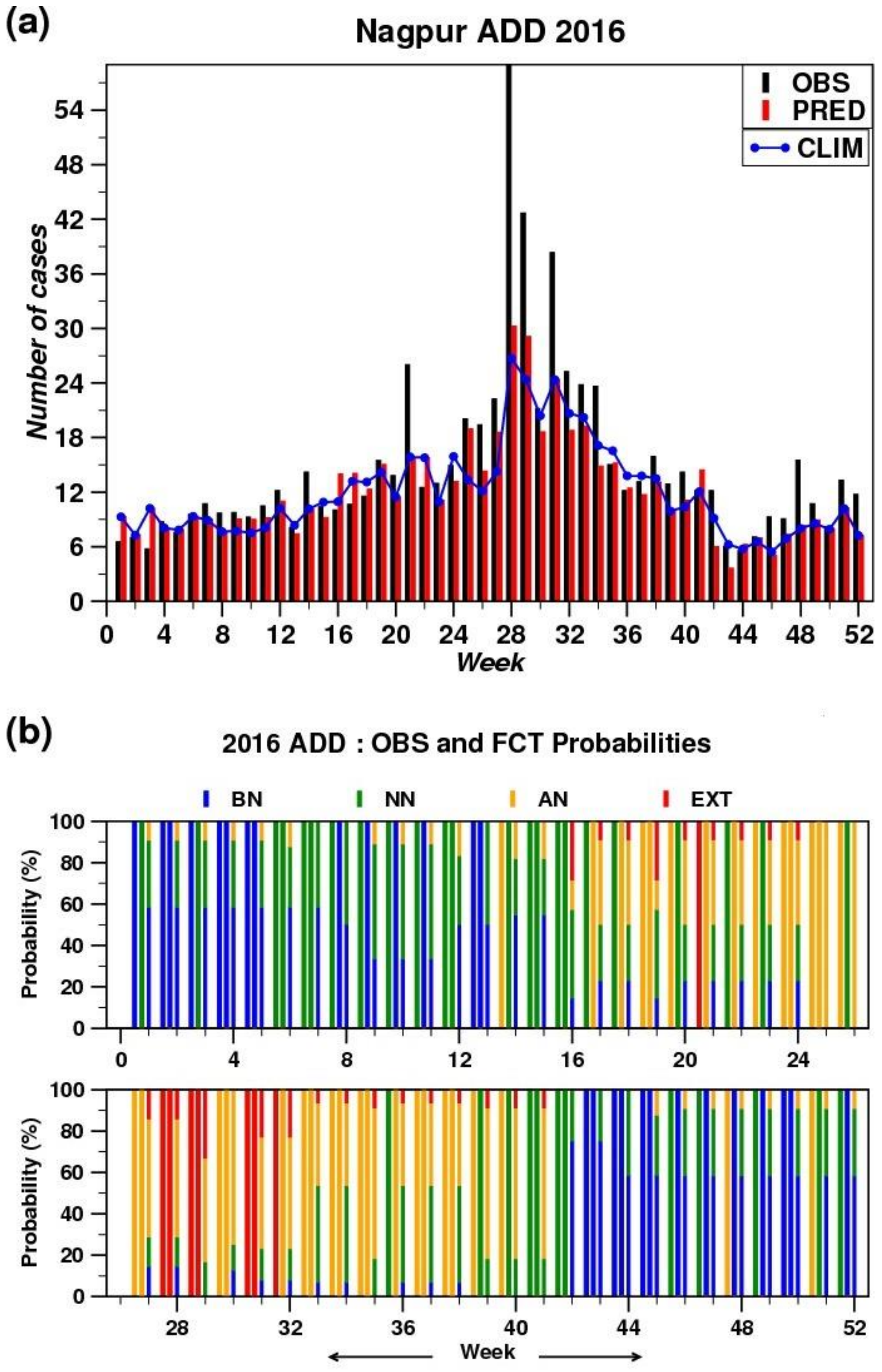

Figure S15: Same as Figure S8, but for ADD during 2016 over Nagpur.

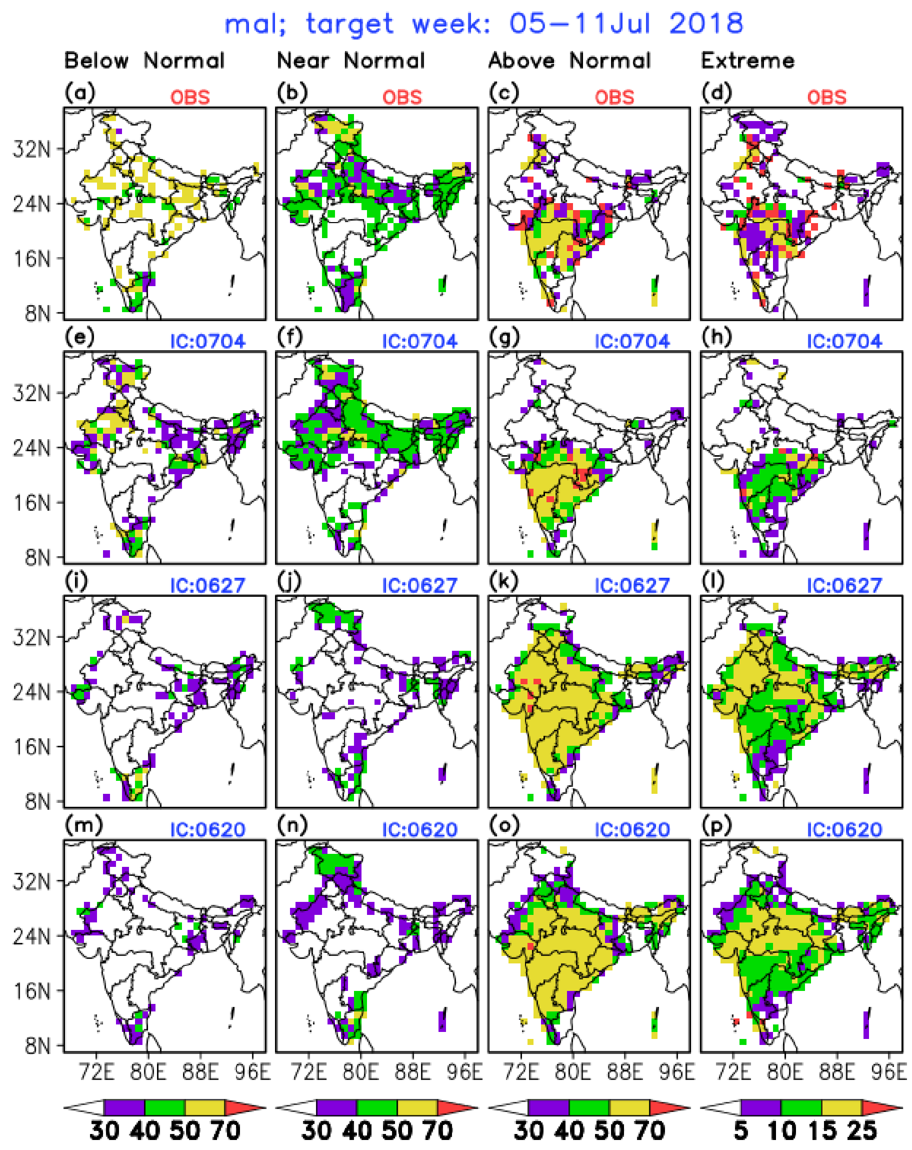

Figure S16: Probabilities of Below Normal (BN), Near Normal (NN), Above Normal (AN) and Extreme (EXT) category of occurrences of **MAL during 05-11 July 2018** for observation (a-d) and forecasts from initial conditions 4th July (e-h), 27th June (i-l) and 20th June (m-p) of 2018. (This map is generated using GrADS (version 2.0.2.oga.1), http://cola.gmu.edu/grads/)

From Figure S16, it is found that, the SOM based early health warning system is able to predict all the categories (specially NN, AN and EXT) reasonably well at least from the nearest two ICs with decreasing probabilities and increasing error with the lead time.

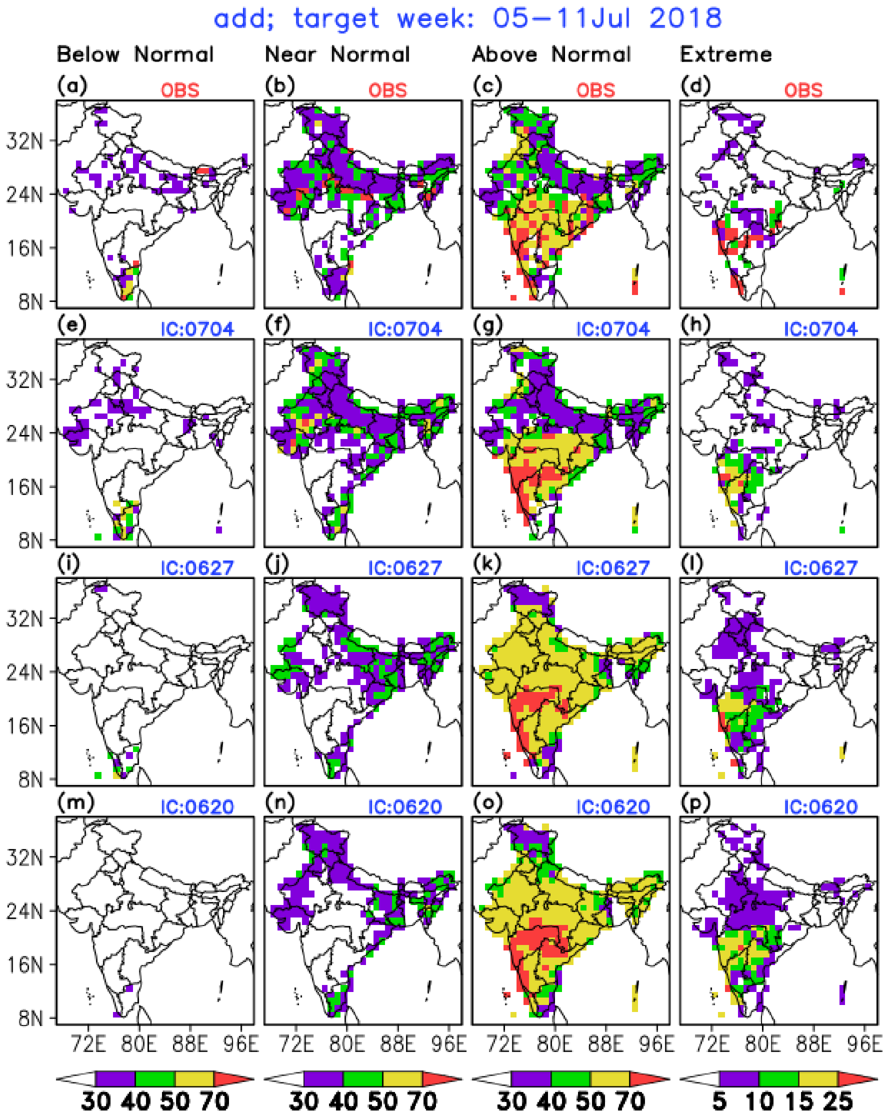

Figure S17: Probabilities of Below Normal (BN), Near Normal (NN), Above Normal (AN) and Extreme (EXT) category of occurrences of **ADD during 05-11 July 2018** for observation (a-d) and forecasts from initial conditions 4th July (e-h), 27th June (i-l) and 20th June (m-p) of 2018. (This map is generated using GrADS (version 2.0.2.oga.1), http://cola.gmu.edu/grads/)

From Figure S17, it can be observed that during this target week most of the places over India, except extreme southern part, experience high chance of AN occurrence of ADD. But over central India and west coast, this probability is much higher (> 50% and somewhere > 70%), and that is well predicted by the nearest three ICs. Though over other parts it over estimates from the longer leads. The EXT category is reasonably well predicted (mainly over central and western India) from all lead times.

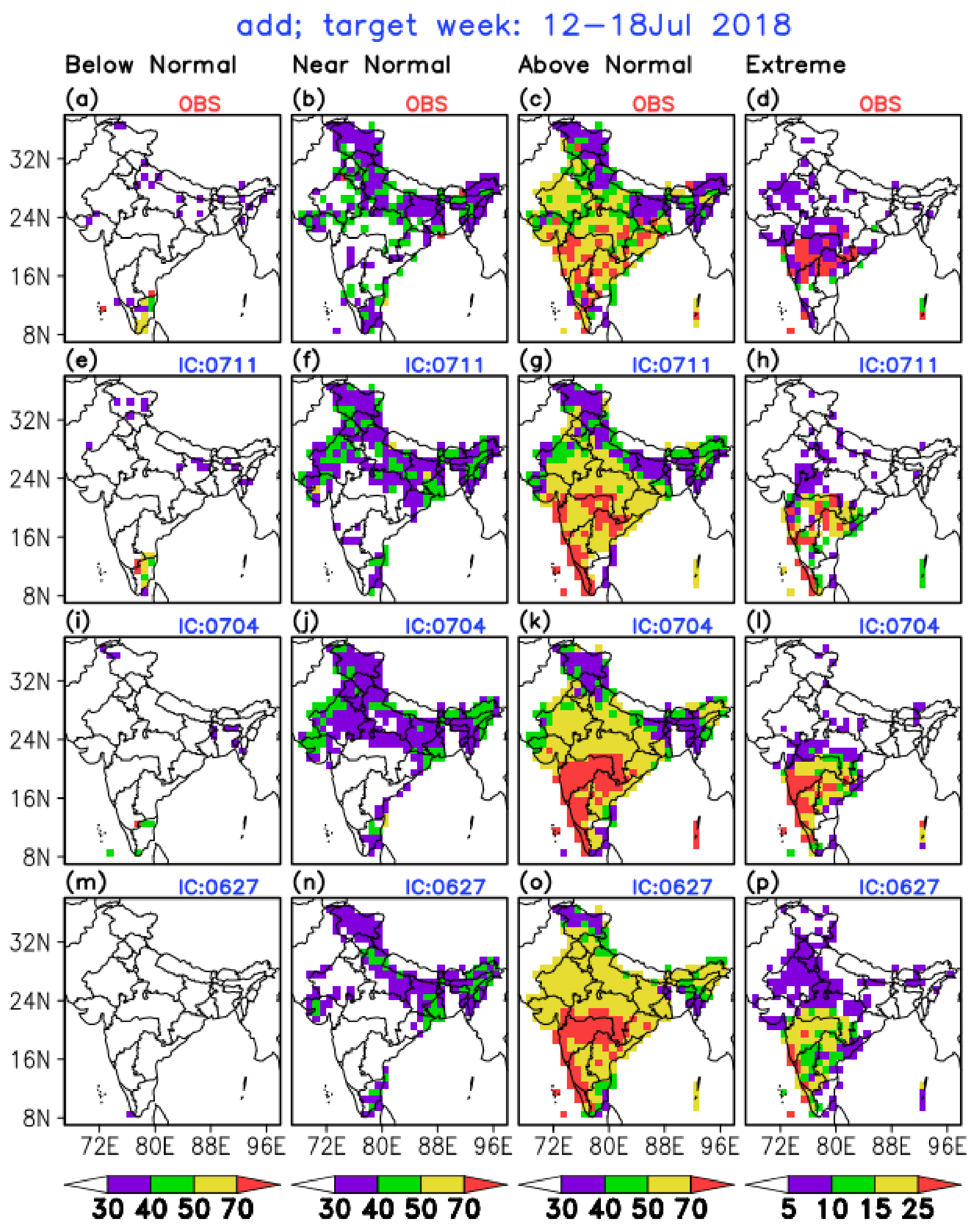

Figure S18: Same as Figure S17, but for **ADD during 12-18 July 2018**. (This map is generated using GrADS (version 2.0.2.oga.1), http://cola.gmu.edu/grads/)

Figure S18 shows the observed and forecasted probabilities (from nearest three ICs) of BN, NN, AN and EXT categories (as mentioned on the image) for the week 12-18 July, 2018 for ADD. In this case also, the prediction system could predict all the categories (especially AN and EXT) reasonably well from all the ICs, though there is an over estimation tendency from the farthest ICs.

Tables

Table S1: Correlation Coefficient (CC) and Root Mean Square Error (RMSE) between observed and predicted (with and without bias correction) Malaria and ADD cases and Brier Skill Scores (BSS) between observed and predicted probabilities for different categories such as Below Normal (BN), Near Normal (NN) and Above Normal (AN). Without bias correction (BC) values are kept inside the brackets. [With R/f, TMx, TMn and humidity as inputs in the SOM clustering]

**Type of Events**	**CC** (without BC)	**RMSE** (without BC)	**BSS**		
			**BN**	**NN**	**AN**
**6x6 SOM**					
Pune Malaria	0.65 (0.54)	5.8 (6.4)	0.30	0.14	0.26
Nagpur Malaria	0.73 (0.62)	15.8 (18.0)	0.27	0.18	0.32
Pune ADD	0.74 (0.65)	4.6 (5.1)	0.26	0.22	0.47
Nagpur ADD	0.72 (0.62)	4.8 (5.5)	0.30	0.13	0.41

Table S2: Correlation Coefficient (CC) and Root Mean Square Error (RMSE) between observed and predicted Malaria and ADD cases and Brier Skill Scores (BSS) between observed and predicted probabilities for different categories such as Below Normal (BN), Near Normal (NN) and Above Normal (AN). Without bias correction (BC) values are kept inside the brackets. [With R/f, TMx and TMn as inputs in the SOM clustering]

**Type of Events**	**CC** (without BC)	**RMSE** (without BC)
**6x6 SOM**		
Pune MAL	**0.68** (0.57)	**5.6** (6.3)
Nagpur MAL	**0.74** (0.63)	**15.4** (17.8)
Pune ADD	**0.72** (0.60)	**4.7** (5.4)
Nagpur ADD	**0.72** (0.62)	**4.8** (5.5)
